# Spectral Precision: Recent Advances in Matrix-Assisted Laser Desorption/Ionization Time-of-Flight Mass Spectrometry for Pathogen Detection and Resistance Profiling

**DOI:** 10.3390/microorganisms13071473

**Published:** 2025-06-25

**Authors:** Ayman Elbehiry, Adil Abalkhail

**Affiliations:** Department of Public Health, College of Applied Medical Sciences, Qassim University, P.O. Box 6666, Buraydah 51452, Saudi Arabia; ar.elbehiry@qu.edu.sa

**Keywords:** MALDI–TOF MS, antibiotic resistance, machine learning, microbial diagnostics

## Abstract

With the global rise in antimicrobial resistance (AMR), rapid and reliable microbial diagnostics have become more critical than ever. Traditional culture-based and molecular diagnostic techniques often fall short in terms of speed, cost-efficiency, or scalability, particularly in resource-limited settings. Matrix-assisted laser desorption/ionization time-of-flight mass spectrometry (MALDI–TOF MS) has emerged as a transformative tool in clinical microbiology. Its unparalleled speed and accuracy in microbial identification, along with expanding applications in AMR profiling, make it a leading candidate for next-generation diagnostic workflows. This review aims to provide a comprehensive update on recent advances in MALDI–TOF MS, focusing on its technological evolution, clinical applications, and future potential in microbial diagnostics and resistance detection. We conducted a critical synthesis of peer-reviewed literature published over the last decade, with emphasis on innovations in sample preparation, instrumentation, data interpretation, and clinical integration. Key developments in AMR detection, including growth-based assays, resistance biomarker profiling, and machine learning-driven spectral analysis, are discussed. MALDI–TOF MS is increasingly deployed not only in clinical laboratories but also in environmental surveillance, food safety, and military biodefense. Despite challenges such as database variability and limited access in low-income regions, it remains a cornerstone of modern microbial diagnostics and holds promise for future integration into global AMR surveillance systems.

## 1. Introduction

The accurate and timely identification of microbial pathogens remains a cornerstone of effective clinical decision-making, particularly in the context of emerging infectious diseases and the growing threat of antimicrobial resistance (AMR). Traditional diagnostic methods—primarily culture-based and biochemical approaches—have long been considered the gold standard, yet they are increasingly criticized for being time-consuming, labor-intensive, and often inadequate for rapid clinical intervention [1,2]. Although molecular techniques such as 16S rRNA gene sequencing, multilocus sequence typing (MLST), and whole-genome sequencing (WGS) offer an exceptional taxonomic resolution and are widely regarded as gold standards for microbial classification, their routine use in diagnostic workflows remains limited [3]. This limitation is largely attributable to high operational costs, complex sample preparation, the need for specialized expertise, and extended turnaround times. These constraints are particularly consequential in the identification of clinically and epidemiologically important microorganisms—such as bacteria, yeasts, and filamentous fungi—where timely and precise detection is essential for initiating appropriate therapy and preventing the spread of infection [4,5]. As a result, there is an urgent need for alternative diagnostic platforms that are rapid, cost-effective, and scalable across diverse clinical, food safety, and environmental settings [2,6,7,8].

In response to this demand, matrix-assisted laser desorption/ionization time-of-flight mass spectrometry (MALDI–TOF MS) has emerged as a transformative tool in clinical microbiology. This technique identifies microorganisms by analyzing the mass-to-charge (*m*/*z*) ratios of ionized biomolecules embedded in a matrix such as α-cyano-4–hydroxycinnamic acid [9,10,11,12,13]. Its ability to generate reproducible protein profiles—or molecular fingerprints—enables accurate species-level identification across a wide range of pathogens [14,15]. Although the initial investment in MALDI–TOF MS instruments is substantial, the platform offers considerable long-term advantages, including ease of operation, a high throughput, rapid turnaround time, and low per-sample cost [16]. Today, MALDI–TOF MS is routinely employed in both clinical and veterinary laboratories and is increasingly replacing traditional culture-based methods [15]. It has demonstrated outstanding performance in the identification of bacteria [17,18,19,20], mycobacteria [21], and fungi [18,22], and is now extending its utility to include viruses [23], protozoans, and arthropods [24,25]. In standard workflows, microbial colonies are transferred onto a target plate, treated with a matrix solution, and analyzed to produce mass spectra, which are then compared against commercial or in-house databases for species-level identification—often achievable in under one hour [26]. This speed is especially critical for immunocompromised or chronically ill patients, where early and accurate diagnosis can significantly influence clinical outcomes [27,28].

Beyond traditional bacteriology and mycology, recent applications have extended the use of MALDI–TOF MS to parasitology and entomology, including the accurate identification of helminths, ticks, and mosquitoes—vectors of significant public health concern. These capabilities offer advantages in epidemiological surveillance and outbreak management, especially in endemic regions or low-resource settings [29,30]. Furthermore, although the small proteome of viruses presents challenges, recent advancements in viral protein enrichment and matrix optimization have enabled the detection and differentiation of viral species via MALDI–TOF MS, particularly for influenza and enteric viruses [31]. These emerging applications highlight the flexibility and scalability of MALDI–TOF MS across microbiological subfields.

Although widely employed, conventional AMR detection methods are limited by time delays and substantial resource requirements. Culture-based approaches may take up to 96 h and require trained personnel and laboratory infrastructure [32,33,34]. These delays can lead to empirical antibiotic use, contributing to treatment failure and increased resistance [35,36]. While molecular methods such as PCR provide faster results, they are limited to predefined genetic targets and often fail to capture phenotypic resistance mechanisms, such as those mediated by efflux pumps. Screening for multiple resistance genes also increases complexity and cost [37]. MALDI–TOF MS presents a promising alternative for AMR detection. It can identify resistance-related protein signatures and enzymatic activity, delivering diagnostic results in as little as 38 min at a cost of approximately USD 0.50 per test [37,38,39].

Recent developments integrating MALDI–TOF MS with ML and artificial intelligence (AI) are now being explored to improve the detection of subtle resistance patterns and enhance predictive modeling, paving the way for personalized and real-time antimicrobial therapy [16]. The diagnostic scope of MALDI–TOF MS, as previously discussed, encompasses a wide array of microbial agents. Its expanding applications continue to demonstrate the method’s versatility in both routine and emerging diagnostic contexts.

## 2. MALDI-TOF MS in Clinical Microbiology: A Practical Overview

### 2.1. Concise History of MALDI–TOF MS

MS originated in the 19th century as a chemical analysis tool, but its application to biological macromolecules became viable only with the development of soft ionization techniques in the 1980s [11]. A breakthrough came with the introduction of MALDI, which enabled the analysis of intact proteins and large biomolecules with minimal fragmentation. In recognition of their contributions to this field, John Fenn and Koichi Tanaka were awarded the Nobel Prize in Chemistry in 2002 for advancing electrospray and soft ionization technologies, which made it possible to analyze biomolecules up to 130 kDa in size [40]. MALDI–TOF MS, which typically uses α-cyano-4–hydroxycinnamic acid as the matrix, is particularly well suited for microbial identification [13]. Although early studies in the 1970s explored MS for bacterial identification, inconsistent results due to interference from culture media hindered its clinical application [41]. The advent of MALDI–TOF technology has resolved many of these limitations by providing a reproducible, robust method for protein analysis in microorganisms [42,43].

Since its broader adoption in clinical microbiology approximately 2009, MALDI–TOF MS has streamlined workflows by allowing direct analysis of microbial colonies with minimal preparation [44]. This advancement has significantly reduced turnaround times and improved the overall efficiency of diagnostic laboratories [27,42,43]. MALDI–TOF MS operates by combining soft ionization with time-of-flight detection. A matrix, typically α-cyano-4–hydroxycinnamic acid, facilitates ionization by absorbing laser energy and transferring it to the analytes [15]. This enables the ionization of intact biomolecules, such as ribosomal proteins, without causing significant fragmentation [45]. Once ionized, the biomolecules are accelerated through an electric field in a vacuum [46]. Their flight time is inversely proportional to their *m*/*z* ratio, generating a distinct spectral fingerprint that can be matched against reference databases for microbial identification [9,10,11,12,13,27].

#### Technical Principles of MALDI–TOF MS

MALDI–TOF MS is based on the generation and analysis of ionized biomolecules, primarily proteins, for microbial identification. The process begins with the preparation of a target sample by mixing a microbial extract with a specialized matrix compound, typically α-cyano-4–hydroxycinnamic acid (HCCA). This matrix facilitates the absorption of ultraviolet (UV) laser energy and promotes the desorption and ionization of sample molecules with minimal fragmentation. Once dried on a target plate, the co-crystallized sample-matrix mixture is irradiated by a UV laser pulse (commonly 337 nm). The matrix absorbs the energy, enabling the ionization of proteins and peptides—usually in the range of 2000–20,000 Daltons. The resulting ion cloud is accelerated into a vacuum flight tube, where ions are separated based on their mass-to-charge (*m*/*z*) ratios. The time required for ions to reach the detector is inversely proportional to the square root of their *m*/*z* values, a principle governed by the equation:

$$t=\frac{L}{\sqrt{2V/m}}$$

where *t* is the time of flight, *L* is the length of the flight tube, *V* is the accelerating voltage, and *m* is the ion mass.

The generated spectra, consisting of mass peaks representing various ribosomal and cytoplasmic proteins, form a unique molecular fingerprint for each microorganism. These spectral patterns are then compared to reference databases (e.g., Bruker Biotyper, VITEK MS) using proprietary algorithms that score the similarity and assign species-level or genus-level identity. Modern MALDI–TOF MS platforms are also capable of handling complex spectra through internal calibration, baseline correction, and noise filtration. Sample preparation methods vary according to organism type: for example, fungi and mycobacteria often require protein extraction protocols using ethanol-formic acid or bead-beating to disrupt tough cell walls. Additionally, new high-resolution systems, such as MALDI–TOF/TOF and MALDI–Orbitrap hybrids, are improving detection sensitivity and post-translational modification analysis. Despite its robust performance, MALDI–TOF MS faces challenges such as ion suppression in mixed cultures, matrix effects, and limited spectral coverage for rare or environmental organisms. To address these limitations, machine learning-driven spectral interpretation and the development of expanded, curated databases are being actively pursued.

### 2.2. Workflow Integration of MALDI–TOF MS at Clinical Laboratories

MALDI–TOF MS identifies microorganisms by measuring the *m*/*z* ratios of ionized biomolecules, producing unique spectral patterns that serve as molecular fingerprints [47,48]. A key component of this process is the matrix, typically a small organic acid, which facilitates soft ionization by absorbing laser energy and transferring it to the analytes. This minimizes molecular fragmentation and preserves the integrity of proteins during ionization. The ionized molecules are accelerated in a vacuum, and their time-of-flight is recorded to determine their respective *m*/*z* values. These data generate a characteristic spectrum that is compared to reference databases for microbial identification [49].

Ribosomal proteins, which are highly conserved and abundantly expressed, are the principal biomarkers detected in microbial spectra [27]. Modern MALDI–TOF MS systems are equipped with extensive spectral libraries, some of which include over 2000 clinically relevant microbial species [27,43]. Spectral comparisons yield identification scores: values above 2.0 generally indicate species-level identification, whereas scores between 1.7 and 2.0 suggest genus-level identification. Commercial platforms such as the Bruker Biotyper and VITEK MS (bioMérieux) have demonstrated up to 99.9% accuracy in species-level identification in clinical settings [50]. While peptide mass fingerprinting can offer deeper diagnostic resolution, its broader application is limited because of the higher cost and complexity of the required instrumentation. Nonetheless, MALDI–TOF MS has been widely integrated into routine clinical workflows because of its speed, accuracy, and cost-effectiveness.

### 2.3. Preparation of Diverse Samples

**Figure 1:**
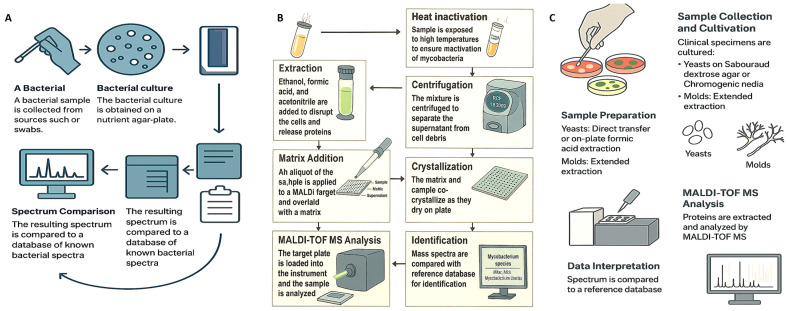
Schematic workflows for microbial identification using MALDI–TOF MS across three organism groups. (**A**) Bacterial workflow: from clinical specimen collection and nutrient agar culture to protein extraction and mass spectral analysis using a bacterial reference database. (**B**) Mycobacterial workflow: incorporating heat inactivation, centrifugation, ethanol-formic acid extraction, matrix application, crystallization, and spectral matching against a mycobacterial-specific library. (**C**) Fungal workflow: covering cultivation on selective media, direct or extended protein extraction, and analysis using a fungal-specific spectral database. Each workflow reflects the unique biological and diagnostic requirements of the respective microorganism.

Effective sample preparation is critical for obtaining accurate and reproducible results with MALDI–TOF MS. In most routine clinical settings, microorganisms are cultured on solid media, and a single colony is transferred to the target plate for analysis. However, certain microorganisms—such as Mycobacterium species and filamentous fungi—require more rigorous extraction protocols to isolate a sufficient protein content for reliable identification [28]. While rapid processing is feasible in urgent clinical scenarios, adequate sample cleanup is essential to reduce interference from host-derived materials and ensure high-quality spectra (Figure 1).

#### 2.3.1. Common Preparation in a Clinical Setting

For many microorganisms, especially common bacterial pathogens, the intact-cell or whole-cell MALDI–TOF MS approach is sufficient [51]. In this method, a microbial colony is applied directly to the MALDI plate and treated with 1 µL of HCCA matrix solution (typically composed of 50% acetonitrile and 2.5% trifluoroacetic acid in water) to facilitate protein extraction and crystallization. However, certain organisms, such as yeasts and Mycobacterium species, possess robust cell walls that hinder protein release and thus require additional extraction steps. In these cases, the ethanol–formic acid protocol is used: the microbial pellet is resuspended in water, mixed with ethanol for inactivation, centrifuged, and subsequently treated with formic acid and acetonitrile before application to the target plate [52].

#### 2.3.2. Preparation of Liquid Samples

Direct identification of pathogens from liquid clinical samples—such as positive blood cultures—requires the removal of interfering substances such as host cells, resins, or charcoal. Among these, blood represents a particularly critical matrix due to the urgency of diagnosing bloodstream infections like sepsis, where delays in pathogen identification can significantly impact patient outcomes. Typically, a two-step centrifugation process is employed: a low-speed spin removes blood cells, followed by high-speed centrifugation of the supernatant to collect microbial cells [53,54]. Mild detergents, such as saponin and sodium dodecyl sulfate (SDS), are often employed to selectively lyse eukaryotic cells while preserving bacterial integrity [54,55]. CE-IVD-labeled commercial kits now enable direct MALDI–TOF MS identification from as little as 1.5 mL of blood culture broth [56]. After separation and formic acid extraction, pathogens can be detected at concentrations as low as 10–10^5^ CFU/mL [57,58]. Sample processing varies by specimen type. In urine, for example, flow cytometry is often used for screening, followed by centrifugation to remove leukocytes and concentrate bacterial cells. Formic acid extraction is then performed before MALDI–TOF MS analysis [58]. For cerebrospinal fluid (CSF), especially in meningitis cases, a common approach involves mixing 500 µL of CSF with 100 µL of 13% SDS, vortexing, centrifugation at 13,000× *g*, and fixation prior to analysis [59,60,61].

#### 2.3.3. Specialized Preparation for Mycobacteria and Fungi

MALDI–TOF MS has substantially shortened the time required to identify Mycobacterium species, which can take weeks to grow via conventional methods. Sample preparation protocols vary among commercial systems. The Bruker Biotyper uses 30 min of heat inactivation at 95 °C, while the VITEK MS protocol by bioMérieux involves vortexing the sample with silica beads in 70% ethanol, followed by chemical extraction using formic acid and acetonitrile. The resulting protein extract is applied to the MALDI target plate and overlaid with matrix solution before analysis [62]. For filamentous fungi, sample preparation depends on the growth conditions and medium. Although liquid culture (e.g., Sabouraud broth) is recommended, solid media such as Sabouraud gentamicin–chloramphenicol agar are also commonly used. After being incubated at 27 °C for 72 h, the fungal mycelium was harvested and subjected to a multistep extraction process. This process typically includes resuspension in sterile water and ethanol, centrifugation, air drying, and sequential treatment with formaldehyde and acetonitrile prior to MALDI–TOF MS analysis [63,64].

## 3. Applications of MALDI–TOF MS for Microbial Identification

MALDI–TOF MS has revolutionized microbial identification over the past two decades, offering unmatched speed, accuracy, and cost-effectiveness compared to traditional phenotypic and molecular diagnostic methods [11,49,65,66,67,68]. By analyzing protein-based mass spectra, this technique enables the rapid and precise detection of a broad spectrum of microorganisms—including bacteria [69], fungi [70,71], parasites [72,73], and even microbial toxins. While initially developed for taxonomic classification, the application range of MALDI–TOF MS has significantly broadened. It now plays a crucial role in identifying AMR mechanisms [74,75] and supports high-throughput diagnostics in diverse disciplines such as virology, mycology, and parasitology [76,77].

In virology, MALDI–TOF MS facilitates the differentiation of viral strains, mutation detection, and identification of pathogens such as influenza and respiratory syncytial viruses. By targeting specific viral proteins, it enhances diagnostic speed and accuracy, surpassing conventional virological techniques in several use cases [77,78]. In mycology, MALDI–TOF MS is widely used for the identification of yeasts and filamentous fungi. More recently, its capacity has expanded to include antifungal resistance profiling, which is particularly important for timely treatment in immunocompromised patients [79]. In parasitology, the technique is gaining traction for the characterization and identification of various protozoan and helminthic parasites [80,81,82,83,84], providing an alternative to labor-intensive microscopic examination and molecular assays. From clinical laboratories to environmental surveillance, food safety testing, and military biodefense, MALDI–TOF MS continues to demonstrate its growing value as a cornerstone of modern microbial diagnostics, enhancing public health monitoring and biothreat preparedness.

### 3.1. Clinical Applications

The traditional identification of bacterial pathogens in clinical samples often relies on metabolic and biochemical profiling, which can take 24 to 48 h to yield results [85]. During this delay, patients are frequently prescribed broad-spectrum empirical antibiotics, which may be ineffective or unnecessary. Consequently, there is a pressing need for rapid, cost-effective diagnostic methods that enable timely, targeted treatment. MALDI–TOF MS has proven to be a valuable tool in this regard, improving antibiotic stewardship and reducing AMR rates [86,87,88]. For example, a study by Clerc et al. reported that MALDI–TOF MS had a positive impact on clinical outcomes in more than 35% of bacteremia cases [89]. Moreover, omitting the centrifugation steps further shortened the hospital stay by approximately two days. In cases of septic shock, this method has contributed to the more precise use of carbapenems, thus reducing the dependence on broad-spectrum antibiotics. MALDI–TOF MS has also demonstrated significant potential in the early identification of sepsis-causing organisms, allowing for effective therapy within 48 h [90]. These improvements are particularly crucial in high-risk environments such as surgical wards, intensive care units, and oncology departments, where delays in diagnosis can increase mortality risk [91].

Historically, microbial identification has been based on phenotypic traits from cultures or biochemical assays—techniques that are too slow for the demands of modern healthcare. Although genetic tools such as 16S and 18S rRNA sequencing and real-time PCR have been available since the 1960s, their complexity and cost limit their routine clinical use [92]. In contrast, MALDI–TOF MS offers immediate species-level identification and has been widely adopted for this purpose. One of the earliest demonstrations of MS-based bacterial differentiation was by Anhalt and Fenselau in 1975 [41]. The introduction of MALDI–TOF MS in the 1990s further improved the resolution and speed of bacterial identification. Subsequent studies have shown its ability to distinguish both Gram-negative and Gram-positive bacteria within minutes [93,94,95,96]. These gains have been supported by advances in sample preparation, spectral database expansion, and software refinement. Today, commercial systems such as VITEK^®^ MS and Bruker’s MALDI Biotyper are used extensively in clinical laboratories, producing species-specific spectral fingerprints for routine diagnostic use [91].

Originally designed for bacterial identification, MALDI–TOF MS has since been extended to include yeasts and filamentous fungi through whole-cell mass fingerprinting. Both major platforms—VITEK^®^ MS and MALDI Biotyper—are FDA-approved for fungal identification. However, diagnostic performance varies on the basis of the fungal species and platform used [71]. For example, the VITEK^®^ MS Knowledge Base v3.0 correctly identified 67% of 319 mold isolates, whereas an updated SARAMIS database achieved a 77% success rate [97]. Variability in identification is often linked to isolate type and sample processing protocols [98,99]. Future database upgrades aim to increase species coverage and improve spectral reliability. Both commercial and custom libraries offer the flexibility to tailor databases to specific institutional needs [71].

In addition to taxonomic identification, MALDI–TOF MS is increasingly being used to detect AMR mechanisms. This method can identify resistance-related proteins, enzymatic modifications, and even antibiotic degradation products. For example, β-lactam antibiotics can detect β-lactamase activity by analyzing the hydrolysis products of β-lactam antibiotics, which exhibit distinctive spectral patterns [100]. In a 2007 study, Camara et al. reported a specific protein peak (~29 kDa) in ampicillin-resistant *E. coli* after incubation with the drug, corresponding to β-lactamase production [101]. Additional studies using SELDI-TOF MS have shown that resistance-associated gene expression can be identified by comparing spectral profiles between susceptible and resistant strains [102]. More recently, modified MALDI target plates—such as those coated with titanium dioxide—have enabled the detection of resistance proteins in intact bacterial cells. This innovation has allowed accurate identification of extended-spectrum β-lactamase-producing *E. coli*, multidrug-resistant *Pseudomonas aeruginosa*, and methicillin-resistant *Staphylococcus aureus* (MRSA) [103].

### 3.2. Food Microbiological Applications

Food safety remains a global public health priority, as contaminated food affects approximately 10% of the world’s population annually, according to the World Health Organization [104]. Contamination can occur at any stage of the food production chain—from processing and packaging to storage and distribution—often due to inadequate hygiene practices or human error [105,106]. While foodborne illness typically presents with gastrointestinal symptoms such as nausea, vomiting, and diarrhea, the consequences can be far more severe, particularly in vulnerable populations [107]. Pathogens such as *Escherichia coli* (*E. coli*), *Staphylococcus aureus*, Salmonella, and *Listeria monocytogenes* can cause illness even at low levels of contamination [108], leading to not only health risks but also substantial economic losses [109]. The detection of foodborne pathogens has traditionally relied on culture-based methods. Although effective, these approaches are slow and often fail to meet the demands of modern food safety protocols [110]. MALDI–TOF MS offers a compelling alternative, providing rapid, high-throughput identification of foodborne bacteria by analyzing their unique protein fingerprints, which can be complemented by ribosomal DNA sequencing for enhanced resolution [111,112]. This integration enables flexible, scalable diagnostics with reduced turnaround times and costs.

To improve detection performance, laboratories are increasingly combining MALDI–TOF MS with advanced technologies such as PCR, immunoassays, next-generation sequencing (NGS), and biosensors. These hybrid approaches significantly reduce the detection time—often to less than 24 h—compared with the 72 h required for traditional methods [113,114]. Among these methods, MALDI–TOF MS is particularly valuable because of its precision, speed, and affordability, and it sometimes even outperforms PCR and NGS in terms of efficiency [11]. It has been successfully employed to detect pathogens such as Salmonella, Campylobacter, and members of the Enterobacteriaceae family across a variety of food matrices. For example, Salmonella Typhimurium has been identified in minced beef [115], whereas *E. coli* and *Campylobacter jejuni* have been detected in traditional foods such as Matazeez [22]. Additional studies have reported the detection of Klebsiella, Enterobacter, and other bacterial contaminants in fish, meat, dairy products, and vegetables from markets in Egypt [116]; vegetables in Beijing [117]; salads, hamburgers, and tortillas in Saudi Arabia [22]; and raw milk from public markets in Turkey [118]. MALDI–TOF MS has also been used to identify *Clostridium difficile* and *Clostridium perfringens* in baby food and infant formulas [119]. Despite some limitations, pairing MALDI–TOF MS with culture-based methods remains a standard practice in food microbiology research [120].

Fungal contamination in food is another significant concern, particularly due to the risk of mycotoxin production. Traditional methods for detecting fungi can take up to one week, delaying critical interventions. To address this, MALDI–TOF MS is now being combined with molecular techniques—such as PCR, immunoassays, and next-generation sequencing (NGS)—to accelerate fungal identification [121,122,123]. The method has proven effective in detecting fungi such as Rhizopus, Aspergillus, Fusarium, and Mucor [22]. In one study, MALDI–TOF MS successfully identified *Aspergillus niger*, *Aspergillus flavus*, Alternaria alternata, Penicillium digitatum, and *Candida albicans* in various food products—including salads, hamburgers, kabsa, and jareesh—from the Al–Qassim region in Saudi Arabia [22]. Further applications include the detection of other fungi, such as *Mucor* [121], foodborne yeasts [124], and fungi present in fermented foods [125]. However, fungal detection via MALDI–TOF MS is still less common than bacterial detection, primarily due to the limited coverage in fungal spectral databases and the need for specialized sample preparation protocols [126].

In immunocompromised individuals, invasive fungal infections represent a significant clinical challenge. For example, species within the Aspergillus genus—such as *A. calidoustus* and *A. lentulus*—are known to exhibit azole resistance, necessitating accurate identification. [127]. MALDI–TOF MS has dramatically improved the detection and differentiation of these morphologically similar strains, reducing misidentification rates from nearly 10% to just over 1% [128]. Additionally, it has enabled the rapid identification of rare fungi such as *Rasamsonia argillacea*, which were often misclassified in the past [97]. In high-risk patients with suspected fungemia, MALDI–TOF MS can quickly identify species such as *Fusarium solani* and *Fusarium verticillioides* from blood cultures. Although antifungal susceptibility testing via MALDI–TOF is still under refinement, studies have shown promising concordance with Clinical and Laboratory Standards Institute (CLSI) guidelines—particularly for caspofungin susceptibility in Candida and Aspergillus species [127].

### 3.3. Environmental Applications

Earth’s ecosystems host an immense diversity of microbial organisms, with hundreds of millions of species identified through metagenomics and other culture-independent techniques [129]. Despite these advances, cultivating many of these microorganisms remains a challenge. Nevertheless, successful cultivation is essential for studying microbial metabolism and for harnessing specific strains for biotechnological applications. While it is unlikely that every member of the microbiome can be grown in the laboratory, replicating the conditions for closely related organisms has made it possible to isolate many novel taxa. MALDI–TOF MS has proven to be a rapid and reliable method for identifying microorganisms from a wide range of environments, including soil, water, hospital surfaces, and even spacecraft [130,131,132,133]. However, the technique’s effectiveness in nonclinical settings is hindered by limitations in current databases, which are heavily skewed toward clinical isolates [130,132]. This bias stems from the underrepresentation of environmental strains in existing spectral libraries and the lack of standardized validation protocols for reference spectra [11]. Expanding these databases is an active area of development to improve MALDI–TOF MS applicability across environmental microbiology.

Multiple reviews have supported the utility of MALDI–TOF mass spectrometry (MS) beyond clinical microbiology. While some works focus solely on clinical diagnostics [9,28], others have explored broader applications, including environmental monitoring and microbial ecology [11,134,135]. For example, Ashfaq et al. [136] demonstrated the method’s potential for identifying novel bacterial species from the rhizosphere and phyllosphere via combined protein profiling and metabolomics, particularly for plant-associated pathogens. Environmental factors can significantly influence microbial phenotypes, occasionally complicating MALDI–TOF MS-based identification. In one study, Niestepski et al. [137] examined *Bacteroides fragilis* from sources such as human and rat feces, hospital wastewater, and both treated and untreated sewage. The identification accuracy was 100% in the fecal samples but decreased to 40% in the treated wastewater and 20% in the untreated wastewater, likely due to increased strain diversity in the environmental samples. These findings emphasize the need for tailored databases that reflect environmental diversity. Similar challenges are found with the *Burkholderia cepacia* complex—a group of phenotypically similar species important in clinical, agricultural, and industrial contexts. Studies by Fehlberg et al. [138], Vicenzi et al. [139], and Furlan et al. [140] revealed that environmental isolates are often misidentified, sometimes confused with unrelated species such as *Ochrobactrum anthropi*. Expanding spectral libraries for these taxa is thus critical for achieving accurate identification.

Nevertheless, successful applications continue to emerge. Hazen et al. [141] used MALDI–TOF MS to differentiate *Vibrio alginolyticus* from other Vibrio species by comparing the protein profiles of environmental and clinical strains. Similarly, Sulaiman et al. [142] reported that the VITEK MS system accurately identified Staphylococcus strains from diverse sources, regardless of their origin. However, misidentification remains a concern—particularly for lesser-studied genera such as *Virgibacillus*, *Bacillus*, *Brachybacterium*, and *Vagococcus* [143,144]. Brauge et al. [144], for example, successfully identified *Virgibacillus halodenitrificans* in seafood and saltwater, highlighting the potential of improved databases for environmental monitoring. To address these issues, several groups have developed custom in-house libraries. Pinar-Mendez et al. [145] created a drinking water database with 319 strains from 96 taxa, increasing the identification accuracy from 54.8% to 76.2%. Similarly, Emami et al. [146] built a spectral library for *Pseudoalteromonas* species in seawater, whereas Fergusson et al. [147] developed a customized database for *Burkholderia*, *Caballeronia*, and *Paraburkholderia*, identifying 39 out of 49 unknown strains at the genus level. These efforts highlight the growing reliability of MALDI–TOF MS for environmental applications—particularly when paired with expanded or tailored databases. Its advantages in terms of speed, cost efficiency, and adaptability position it as a valuable tool for exploring microbial diversity across ecosystems.

### 3.4. Military Applications

The need for rapid and reliable identification of biological threats became especially apparent following the 1991 disclosure of Iraq’s biological weapons program. Historical incidents such as the anthrax outbreak in Sverdlovsk, which caused 138 fatalities, and the intentional Salmonella contamination in Oregon, which affected more than 750 people, underscore the importance of advanced biosurveillance systems [112,148]. The confiscation of dangerous biological materials—such as anthrax vaccines, *Yersinia pestis* cultures, and ricin—by U.S. military personnel further highlights the risks posed by biological warfare agents (BWAs). In response, military and security agencies have accelerated the development of tools capable of detecting such agents within minutes of exposure. While traditional methods for identifying biothreat organisms—such as phenotypic, genotypic, and immunologic assays—can be accurate, they are often too slow, complex, or hazardous for frontline deployment. MALDI–TOF MS has emerged as a practical and efficient alternative, offering rapid and precise identification of high-risk pathogens, including Brucella spp., *Coxiella burnetii*, *Bacillus anthracis*, and *Francisella tularensis* [149,150,151,152]. Its speed and minimal sample preparation make it especially useful in field settings and high-containment laboratories.

One notable challenge in BWA detection involves the identification of bacterial spores, which differ significantly from their vegetative forms in terms of protein composition and physical properties [153]. To address this, Jeong et al. developed an in situ MALDI–TOF MS system capable of high-throughput identification of aerosolized Bacillus spores, supported by a specialized spectral database and matching algorithm [154]. Their system was designed to target aerosol particles in the 2–10 µm range—ideal for detecting airborne BWAs. Importantly, this platform allows real-time analysis without the need for time-consuming sample preparation. By identifying unique biomolecular markers, the system enables rapid, accurate detection of spores and supports timely responses in potential bioterrorism scenarios [155]. Overall, MALDI–TOF MS enhances biosurveillance by enabling fast, high-confidence identification of pathogens associated with biological warfare. Its portability, automation potential, and compatibility with custom threat databases make it a powerful tool in both military defense and civilian emergency preparedness.

### 3.5. Detection of AMR by MALDI–TOF MS

#### 3.5.1. Workflow Overview

**Figure 2:**
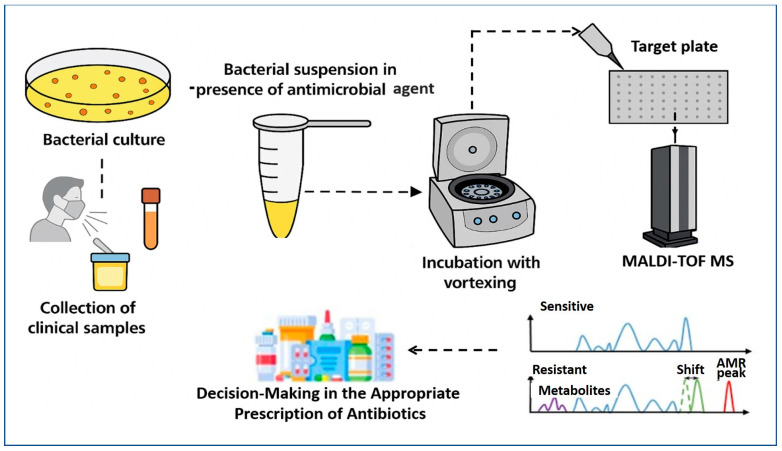
Schematic representation of AMR detection workflow using the MALDI–TOF MS approach. The process involves sample preparation from bacterial isolates, acquisition of mass spectra with or without antibiotic exposure, and analysis of spectral patterns. Sensitive strains show typical protein profiles, while resistant strains exhibit distinct spectral features such as metabolite peaks, mass shifts, or specific AMR-associated peaks. These differences enable rapid identification and resistance profiling based on mass spectrometry data.

MALDI–TOF MS has emerged as a transformative tool for the rapid detection of AMR, extending far beyond its initial role in species identification. The core principle involves detecting metabolic or proteomic changes induced by antibiotic exposure in microbial cultures. As described by Idelevich et al. [74], the standard workflow (Figure 2) begins with the collection of clinical specimens—such as blood, urine, or respiratory tract samples—which are cultured to isolate the target organism. A standardized bacterial suspension is then prepared and divided into two aliquots: one exposed to a specific antimicrobial agent, and the other serving as an antibiotic-free control. Following a short incubation period, typically between 2 and 4 h, both aliquots are subjected to MALDI–TOF MS analysis.

The comparative analysis of spectra from treated and untreated samples can reveal key resistance indicators. In susceptible strains, antibiotic-induced metabolic suppression results in reduced biomass or altered protein profiles, leading to diminished or shifted spectral peaks. Conversely, resistant strains continue active protein synthesis, producing characteristic spectra that remain comparable to controls. Notably, Sparbier et al. [156] demonstrated this principle in detecting β-lactamase activity by identifying hydrolyzed β-lactam antibiotic products directly within the mass spectra. This phenotypic method enables direct inference of resistance without requiring genetic amplification or sequencing, thereby offering a clinically actionable turnaround time of just a few hours [12]. Nevertheless, successful implementation depends on well-curated spectral libraries, optimized protocols for different antibiotic classes, and robust quality control measures.

To enhance the diagnostic potential of MALDI–TOF MS, several recent studies have integrated ML algorithms into the analytical pipeline. López-Cortés et al. [157] developed a predictive model using spectral data for *E. coli*, *Staphylococcus aureus*, and *Klebsiella pneumoniae*. Their ML framework, which employed support vector machines and random forest classifiers, achieved high accuracy in detecting resistance across multiple antibiotic classes. This integration not only improved diagnostic performance but also reduced interpretation time, making the approach more suitable for routine clinical use. In a complementary study, Astudillo et al. [158] explored multi-label classification techniques to predict resistance to several antibiotics simultaneously using raw MALDI–TOF MS data. This method proved particularly effective in profiling multidrug-resistant organisms and provided a scalable framework for high-throughput AMR surveillance. Their findings emphasize the feasibility of leveraging complex spectral data to inform antimicrobial treatment decisions more holistically.

The importance of large-scale datasets in enhancing MALDI–TOF-based AMR detection was highlighted by Ren et al. [159], who constructed an extensive spectral database encompassing over 300,000 clinical isolates linked to more than 750,000 resistance phenotypes. By training their models on such a robust dataset, the authors demonstrated the potential of big data in identifying resistance mechanisms across diverse bacterial species. Their study underlines how comprehensive data integration can significantly increase the reliability and clinical relevance of ML-assisted MALDI–TOF MS applications. Additionally, Lin et al. [160] applied a similar approach in the context of food safety. Their study focused on detecting antibiotic-resistant *E. coli* in food processing environments by combining MALDI–TOF MS with real-time ML models. The system demonstrated excellent performance in both speed and sensitivity, showcasing its utility beyond clinical diagnostics and highlighting its role in One Health surveillance efforts.

The effective implementation of MALDI–TOF MS for AMR detection depends on well-curated spectral libraries, standardized sample preparation, and strict quality control to minimize variability. Key variables—such as incubation time, bacterial load, and ionization conditions—must be tightly controlled. As data complexity increases, integrating ML has become essential to enhance accuracy and automate interpretation. Together, these advances are transforming MALDI–TOF MS into a fast, reliable tool for guiding targeted antimicrobial therapy and stewardship.

#### 3.5.2. Comparison of MALDI–TOF MS with Other AMR Detection Techniques

MALDI–TOF MS has become an increasingly valuable diagnostic tool, initially revolutionizing bacterial identification and now expanding into AMR detection. To contextualize its clinical utility, a comparative overview with established AMR detection methods is summarized in Table 1. Compared with conventional phenotypic assays—such as broth microdilution or disk diffusion—MALDI–TOF MS offers a markedly reduced turnaround time, delivering resistance-related results within hours after culture, while traditional phenotypic methods typically require 18–24 h and manual interpretation under standardized conditions [161]. Florio et al. [162] highlighted this rapidity as one of MALDI–TOF MS’s greatest clinical advantages. Molecular methods such as PCR and WGS provide high sensitivity and specificity for detecting known resistance genes, but are limited by their reliance on prior genetic knowledge. These techniques may miss novel or phenotypically expressed resistance mechanisms, and they often require more time, cost, and advanced infrastructure [163,164].

By contrast, MALDI–TOF MS is cost-effective, reagent-free, and readily adaptable to high-throughput workflows. Its expanding application in AMR detection is driven by its ability to identify resistance-associated proteomic signatures. When paired with ML algorithms, MALDI–TOF MS can discern subtle spectral differences that correspond with resistance phenotypes, allowing rapid, phenotypic-based susceptibility prediction [165]. Weis et al. [37] demonstrated the power of combining MALDI–TOF MS with ML to predict AMR from proteomic profiles with high accuracy. Nevertheless, MALDI–TOF MS does have limitations—particularly in detecting resistance mechanisms not expressed at the protein level or those present in low abundance [166]. Rodriguez-Sánchez et al. [167] reported that although database expansion and improved spectra processing enhance MALDI–TOF MS performance, it cannot yet replace comprehensive genotypic or functional phenotypic assays. Therefore, MALDI–TOF MS should be considered a complementary tool that augments—but does not fully substitute—conventional methods in AMR diagnostics and surveillance.

**Table 1:** Comparative overview of AMR detection methods including MALDI–TOF MS and their sample preparation principles across microbial groups.

Method	Principle	Advantages	Limitations	References
MALDI–TOF MS	Protein fingerprinting with mass spectrometry to detect resistance-related biomarkers or profiles.	Rapid (<1 h), high-throughput, cost-effective after setup, minimal reagent use.	Requires extensive spectral databases, limited direct resistance marker detection, needs ML for improved accuracy.	[74,156]
PCR-based Methods	Amplification of known resistance genes (e.g., *mecA*, *blaKPC*).	High sensitivity and specificity for known genes, relatively quick.	Cannot detect unknown or novel resistance mechanisms, prone to contamination.	[163]
Whole Genome Sequencing (WGS)	Comprehensive sequencing of bacterial genome to identify known and novel AMR genes.	High resolution, detects all known/novel genes, useful for epidemiology.	Expensive, requires bioinformatics expertise, longer turnaround.	[164]
Phenotypic Methods (e.g., Broth Microdilution)	Culture-based growth inhibition assays in the presence of antibiotics.	Gold standard; detects functional resistance.	Slow (16–24 h or more), labor-intensive, not suitable for all clinical workflows.	[168]
Microarray-based Methods	Hybridization-based detection of multiple AMR genes.	Multiplex capability; relatively rapid.	Limited to probe-targeted genes, moderate cost, interpretation complexity.	[169]

#### 3.5.3. Advanced Approaches for AMR Detection

AMR poses a significant global health threat. In 2019, AMR was directly responsible for approximately 1.27 million deaths worldwide, with projections estimating up to 10 million annual deaths by 2050 if effective interventions are not implemented [170,171,172]. These alarming figures underscore the urgent need for rapid and accurate diagnostic methods to guide appropriate antimicrobial therapy. Traditional antimicrobial susceptibility testing (AST) methods, such as broth microdilution and disk diffusion, remain the clinical gold standards but are time-consuming, often requiring 18–24 h to yield results [173,174]. The integration of MALDI–TOF MS into clinical microbiology laboratories has revolutionized microbial identification and opened avenues for rapid AMR detection. MALDI–TOF MS can detect resistance-associated proteomic patterns, allowing for the inference of resistance phenotypes directly from protein fingerprints [175]. This capability facilitates earlier initiation of targeted therapy, supporting antimicrobial stewardship [176].

However, not all resistance mechanisms produce detectable protein-level changes within the spectral range of standard MALDI–TOF instruments [174]. Moreover, subtle biochemical shifts may evade detection, especially in cases involving low-abundance resistance determinants. Rodríguez-Sánchez et al. [167] emphasized that despite advances in spectral resolution and database enrichment, some resistance mechanisms remain challenging to identify due to species-specific variability and incomplete spectral libraries [177]. Recent innovations—such as the DRIAMS database—are helping address these challenges by linking large-scale MALDI–TOF spectra to corresponding resistance phenotypes. These resources enable the training of more robust ML algorithms capable of predicting AMR with greater accuracy and scalability [178,179,180]. By analyzing bacterial protein production in response to antibiotic exposure, MALDI–TOF MS-based AST, on the other hand, can yield results within 2–3 h.

A prominent example is the MBT-ASTRA assay, which calculates relative growth (RG) by comparing spectral peak intensities—quantified as the area under the curve (AUC)—between treated and untreated bacterial cultures. An RG near zero indicates susceptibility, whereas a value near one suggests resistance [181]. This assay enables clinicians to obtain AST results rapidly, enhancing the speed and precision of therapeutic decisions. MBT-ASTRA calculates relative bacterial growth by comparing the area under the curve (AUC) of spectral peaks from cultures exposed to antibiotics (AUC_BAC+ATB_) with those from untreated controls (AUC_BAC_). The ratio RG = (AUC_BAC+ATB_)/(AUC_BAC_) serves as a marker of susceptibility: an RG close to zero suggests that the antibiotic effectively inhibited bacterial growth, whereas an RG near one indicates continued growth despite antibiotic exposure [182]. This approach enables rapid AST results within 2–3 h, significantly enhancing the speed of clinical decision-making [181]. However, MBT-ASTRA currently requires proprietary software and advanced user expertise, limiting its availability in some settings [183].

Other MALDI–TOF MS–based AMR detection approaches focus on identifying antibiotic degradation products, such as those produced by β-lactamases, or observing shifts in protein profiles associated with exposure to antibiotics such as carbapenems [60,184,185,186]. These functional assays offer direct phenotypic evidence of resistance and can be completed within hours—far faster than standard AST methods. Together, these developments position MALDI–TOF MS as a powerful platform for rapid resistance profiling. As research continues to refine spectral interpretation and expand data integration, the technique is increasingly capable of supporting clinical decision-making in the era of multidrug-resistant infections.

#### 3.5.4. Growth-Based Detection

Growth-based detection methods assess AMR by monitoring bacterial proliferation in the presence of antibiotics. If an organism continues to grow despite antibiotic exposure, it is considered resistant. Matrix-assisted laser desorption/ionization time-of-flight mass spectrometry (MALDI–TOF MS) has been adapted to capture this dynamic by detecting changes in protein expression during short incubation periods, enabling rapid susceptibility testing [187]. One innovative technique involves the use of isotopically labeled amino acids to track bacterial protein synthesis. In this method, bacteria are cultured in media containing heavy isotopes—such as labeled lysine—along with antibiotics. After incubation, the mass shift in peptide peaks reveals whether protein synthesis, and thus growth, occurred. For example, Sparbier et al. [156] utilized this approach to differentiate methicillin-resistant *Staphylococcus aureus* (MRSA) from susceptible strains by detecting isotope-labeled peaks following oxacillin exposure. A reduction in the labeled peak intensity in susceptible strains reflected the antibiotic-induced inhibition of protein synthesis. In addition to β-lactam resistance, MALDI–TOF MS has shown promising results in detecting resistance to colistin, a last-resort antibiotic. Li et al. developed a rapid MALDI–TOF MS–based assay capable of distinguishing colistin-resistant and susceptible *E. coli* strains by comparing spectral growth profiles in the presence and absence of colistin. Their approach, which integrated a custom R-based algorithm, achieved 97.4% sensitivity and 88.2% specificity across 128 clinical isolates [188].

Another method, known as the direct-on-target microdroplet growth assay (DOT-MGA), was introduced by Idelevich et al. [74]. In this approach, small volumes of bacterial suspension—typically below the detection threshold of standard MALDI–TOF MS—are incubated with and without antibiotics directly on the MALDI plate. After 3–4 h of incubation, spectral analysis was used to determine whether bacterial growth occurred. The presence of detectable spectra in the antibiotic-exposed sample indicates resistance, whereas the absence of detectable spectra implies susceptibility. Wang et al. [189] used DOT-MGA to estimate the minimum inhibitory concentration (MIC) of carbapenems in *Klebsiella pneumoniae*, correlating MIC values with the presence of carbapenemase genes. Quantitative MALDI–TOF MS methods have also been employed to measure bacterial growth by comparing peak intensities before and after antibiotic exposure. These protocols involve lysing bacterial cells, mixing them with internal standards, and analyzing the peak area as a proxy for growth [190,191]. In a study involving 94 *K. pneumoniae* isolates, meropenem resistance at 8 µg/mL was detected within one hour, achieving 97.3% sensitivity and 93.5% specificity compared with standard Etest results [181]. Similar success has been demonstrated for detecting resistance to cefotaxime, meropenem, and tobramycin in *E. coli*, *P. aeruginosa*, and *A. baumannii* [190].

MALDI–TOF MS has also been adapted to identify resistance in slow-growing organisms such as *Mycobacterium tuberculosis*. Quantitative methods have enabled earlier detection of resistance to clarithromycin and rifabutin in both *M. tuberculosis* and nontuberculous mycobacteria—sometimes up to one week before conventional AST methods [191]. However, for other agents, such as rifampin, isoniazid, linezolid, and ethambutol, no significant time advantage has been reported. A noteworthy advancement comes from the work of Furniss et al. [192], who used the MALDI Biotyper Sirius system to rapidly detect colistin resistance in *E. coli*. Their method focused on the identification of lipid A modification—hallmarks of colistin resistance—directly from intact bacteria. This approach yielded high accuracy and could be completed within hours, further demonstrating the role of MALDI–TOF MS in direct phenotypic resistance testing.

Similarly, Li et al. [193] developed a MALDI–TOF MS–based workflow integrated with a custom R-based algorithm to detect colistin resistance in clinical *E. coli* isolates. By comparing spectral growth profiles in the presence and absence of colistin, the method achieved 97.4% sensitivity and 88.2% specificity across 128 isolates, demonstrating robust performance for real-world diagnostics. As colistin resistance continues to rise—driven in part by the spread of plasmid-mediated mcr genes—accurate, rapid detection platforms like MALDI–TOF MS are essential in the ongoing battle against multidrug-resistant pathogens.

#### 3.5.5. Detection of Specific Resistance Biomarkers

Another promising strategy for AMR detection via MALDI–TOF MS involves identifying specific biomarkers—distinct mass spectral peaks associated with resistant phenotypes. By comparing spectra from resistant and susceptible strains, researchers have pinpointed diagnostic peaks serving as molecular indicators of resistance [194,195,196]. For example, Du et al. [194] detected a unique mass peak at 2413 *m*/*z* that is consistently present in methicillin-resistant *S. aureus* (MRSA) strains but absent in methicillin-susceptible *S. aureus* (MSSA). This peak was later linked to the expression of PSM-mec, a peptide encoded within certain SCCmec elements responsible for methicillin resistance, as demonstrated by Josten et al. [195]. In a study involving 220 MRSA isolates, a peak near 2415 *m*/*z* was consistently detected, reinforcing its utility as a resistance biomarker. However, this approach has limitations: it is specific to MRSA strains harboring SCCmec types II, III, or VIII. Therefore, its effectiveness diminishes in regions such as the United States and Latin America, where PSM-mec-negative clones like CC8-MRSA-IV are more prevalent.

Biomarker-based MALDI–TOF MS has also been applied to detect *Klebsiella pneumoniae* carbapenemases (KPCs). Lau et al. [196] identified a specific peak at 11,109 Da linked to the bla_KPC-3 gene located on the pKpQIL plasmid in carbapenem-resistant *K. pneumoniae*. While this discovery enabled targeted detection, the method’s reliance on manual interpretation and potential plasmid variability limited its diagnostic robustness. To streamline the process, Cordovana et al. [197] incorporated a KPC-specific algorithm into Bruker MALDI Biotyper software version 4.1, allowing for automated detection during routine species identification. However, further evaluation by Figueroa-Espinosa et al. [198] revealed the 11,109-Da peak in only 32% of bla_KPC-2–positive strains, indicating variable detectability. Interestingly, these researchers observed a more consistent peak at 28,544 Da, although it lies outside the instrument’s standard detection range, presenting a challenge for clinical use. Despite these challenges, biomarker-based subtyping continues to have diagnostic value. Cordovana et al. [199] demonstrated that MALDI–TOF MS subtyping could reduce diagnostic turnaround times by up to 24 h for KPC-producing *K. pneumoniae* and by 48 h for MRSA and carbapenem-resistant *Bacteroides fragilis*. Moreover, the same KPC-associated peak identified in *K. pneumoniae* was also found in other Enterobacterales, suggesting possible horizontal gene transfer of resistance determinants.

In another application, Chang et al. [200] proposed a novel approach for detecting β-lactamase-related resistance in *Acinetobacter baumannii*. They utilized detonation nanodiamonds (DNDs) to concentrate carbapenemase-associated proteins, significantly enhancing detection sensitivity. Among various tested conditions, extraction with 0.5% trifluoroacetic acid yielded the strongest ion signals. Their analysis of 66 clinical isolates (51 resistant; 15 susceptible) revealed a specific peak at approximately 40,279 *m*/*z* corresponding to an apparent diffusion coefficient (ADC)-family extended-spectrum β-lactamase. This biomarker achieved 96% sensitivity and 73% specificity compared with imipenem MIC testing—and 100% agreement with PCR—highlighting its potential for rapid, substrate-free AMR detection. Collectively, these studies underscore the capability of MALDI–TOF MS to detect specific resistance-related biomarkers. While limitations persist—such as instrument sensitivity and geographic variability in resistance genotypes—biomarker detection remains a rapid, cost-effective strategy for guiding antimicrobial therapy.

#### 3.5.6. Next-Generation Analytical Approaches for AMR Detection via MALDI–TOF MS

Advances in matrix-assisted laser desorption/ionization time-of-flight mass spectrometry (MALDI–TOF MS) have revolutionized the field of microbial diagnostics, offering rapid and high-throughput identification of pathogens. However, its application in antimicrobial susceptibility testing (AST) has been historically constrained by its reliance on the detection of known resistance biomarkers [74,201]. This limitation poses challenges in clinical implementation due to a reduced sensitivity to low-abundance proteins, complexities in interpreting mass spectral data, and incomplete reference databases that lack coverage of all pathogen–antibiotic combinations [184]. To overcome these challenges, machine learning (ML) has emerged as a transformative analytical tool. ML algorithms are capable of identifying intricate, multidimensional patterns within mass spectra that may not be apparent to human analysts. When integrated with MALDI–TOF MS, ML can significantly enhance sensitivity, reduce false-positive rates, and broaden the spectrum of detectable resistance mechanisms.

Tools such as ClinProTools and flexAnalysis version 2 (Bruker Daltonics, Bremen, Germany) already facilitate this integration. ClinProTools, for example, employs various classification algorithms—including neural networks, genetic algorithms, and random forests—to correlate spectral patterns (within the 2000–20,000 *m*/*z* range) with phenotypic resistance profiles [187,202]. In non-clinical settings, the application of ML-enhanced MALDI–TOF MS is also gaining momentum. Lin et al. [160] successfully applied a random forest model to spectral data obtained from *Escherichia coli* strains isolated from food processing environments. Their model accurately predicted resistance profiles for multiple antibiotics, achieving validation accuracies between 67% and 97%. However, limitations were noted in predicting fluoroquinolone resistance, highlighting the need for continued optimization in algorithm design and dataset expansion.

In the clinical setting, Lopez-Cortés et al. [157] employed multiple ML models—such as support vector machines, logistic regression, and CatBoost—to predict AMR in *Staphylococcus aureus*, *E. coli*, and *Klebsiella pneumoniae* using VITEK^®^ MALDI–TOF spectra (bioMérieux, Marcy-l’Étoile, France). Their use of transfer learning allowed for the integration of data from different mass spectrometry platforms. Among the models, CatBoost yielded the highest performance for *E. coli*, achieving an area under the receiver operating characteristic curve (AUROC) of 0.91 and an F1 score of 0.78. Furthermore, SHAP (SHapley Additive exPlanations) values helped identify key spectral features that contributed most to model predictions, enhancing transparency and interpretability.

Building on this momentum, Ren et al. [159] developed ML models for Staphylococcus epidermidis, utilizing large-scale spectral datasets to predict resistance phenotypes. Their optimized models achieved AUROC values ranging from 0.80 to 0.95 and AUPRC up to 0.97. Notably, they used SHAP values to validate that predictive performance was driven by biologically relevant protein signals rather than background noise, reinforcing the validity of this ML-enhanced approach. Similarly, Feucherolles [203] demonstrated the successful use of MALDI–TOF MS combined with ML for screening AMR in *Campylobacter* spp. isolated from food sources. Their work emphasizes the utility of these methods beyond clinical labs, offering promising applications in food safety and One Health surveillance strategies.

**Figure 3:**
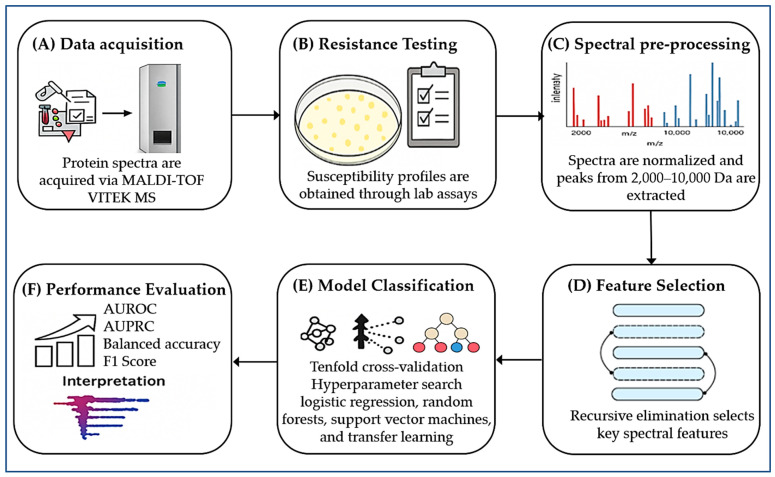
Workflow for predicting AMR using MALDI–TOF MS and ML integration. (**A**) Protein spectra acquisition using the MALDI–TOF VITEK MS system (bioMérieux). (**B**) Antimicrobial susceptibility testing (AST) to generate phenotypic resistance profiles. (**C**) Spectral preprocessing: normalization and extraction of peaks between 2000 and 10,000 Da. (**D**) Feature selection Via recursive elimination to isolate key spectral features. (**E**) Classification using ML models (logistic regression, random forest, support vector machine, transfer learning), optimized by tenfold cross-validation. (**F**) Performance evaluation with AUROC, AUPRC, balanced accuracy, and F1 score; SHAP analysis for identifying key features driving resistance prediction.

Moreover, De Waele et al. [170] introduced an ML-based antimicrobial drug recommender system powered by MALDI–TOF MS. Their novel dual-branch learning model improved prediction accuracy and allowed for efficient adaptation across multiple clinical laboratories. This work exemplifies how next-generation ML pipelines can support real-time clinical decision-making in infectious disease management. In summary, the integration of ML with MALDI–TOF MS is rapidly redefining the diagnostic landscape for AMR (Figure 3). These emerging approaches address the limitations of conventional phenotypic and genetic tests by leveraging high-dimensional spectral data for accurate and rapid resistance profiling. As validated databases and computational infrastructures expand, these tools are poised to become indispensable components of precision diagnostics and antimicrobial stewardship programs.

Complementing these algorithmic advances, hardware innovations have also pushed the boundaries of MALDI–TOF MS. Integrating MALDI–TOF MS with high-resolution mass analyzers such as Orbitrap systems improves the detection of subtle proteomic shifts and low-abundance biomarkers [204]. This hybrid approach enhances strain-level resolution, which is crucial for distinguishing closely related pathogens that differ in resistance mechanisms [201]. These technologies elevate MALDI–TOF MS from species identification to sophisticated resistance profiling, laying the groundwork for precision microbiology. Additionally, cloud computing and federated learning redefine how spectral data are used in AMR surveillance. Federated learning models enable the training of decentralized ML algorithms without centralizing sensitive clinical data, promoting both data privacy and large-scale collaboration. This approach supports real-time, cross-institutional monitoring of AMR trends and facilitates model deployment without extensive site-specific calibration. Reviews by Nguyen et al. [205], Prayitno et al. [206], and Li et al. [207] underscore the potential of federated learning in advancing decentralized, privacy-preserving diagnostics.

Despite these promising developments, key barriers persist. High-resolution mass analyzers, such as Orbitrap systems, remain cost-prohibitive for many laboratories—particularly in low- and middle-income countries—due to their high purchase price, substantial maintenance costs, and need for specialized personnel [208]. These instruments typically range from USD 400,000 to over USD 1 million, making them inaccessible to most institutions without significant capital investment [208]. Similarly, ML-based diagnostic tools require extensive external validation to avoid overfitting, especially when trained on biased or homogeneous datasets [170]. Without adequate oversight, such models may misclassify cases involving novel or rare resistance mechanisms, highlighting the continued necessity of phenotypic testing to support diagnosis and resistance monitoring—particularly in complex clinical scenarios or when new antimicrobial agents are deployed [209]. To safely integrate these next-generation analytical technologies into routine diagnostics, coordinated efforts are required in areas such as data standardization, regulatory harmonization, and cost–benefit analysis. The convergence of high-resolution instrumentation, machine intelligence, and federated data infrastructures has the potential to transform MALDI–TOF MS from a species identification tool into a foundational platform for personalized medicine, antimicrobial stewardship, and global health surveillance [210].

Table 2 summarizes the main strengths, limitations, clinical implications, and future directions of ML-enhanced MALDI–TOF MS for AMR detection. The “Details” column includes both findings drawn from published studies and insights synthesized from the broader discussion presented in Section 3.5.5 and Section 3.5.6. Literature-supported points are cited accordingly, while interpretive conclusions based on the present analysis are marked with an asterisk (*), ensuring clarity and transparency. These ML–driven strategies underscore the ongoing technological evolution of MALDI–TOF MS in AMR diagnostics, which is further detailed in the following section on recent analytical innovations.

#### 3.5.7. Technical Enhancements in MALDI–TOF MS for AMR Detection

In recent years, technical innovations have significantly improved the sensitivity and specificity of MALDI–TOF MS for AMR detection. These enhancements address previous limitations related to complex spectra, low-abundance biomarkers, and mixed microbial populations. One notable advancement is the use of stable isotope labeling, which enhances differentiation between resistant and susceptible strains by tracing metabolically labeled peptides following antibiotic exposure. This approach improves the detection of phenotypic responses at the protein level and allows earlier inference of resistance patterns. For example, Paul et al. [211] demonstrated the utility of this method for rapid detection of fluconazole resistance in *Candida tropicalis* using MALDI–TOF MS and deuterium-labeled glucose to track metabolic activity.

Another key development is the integration of high-resolution mass analyzers such as the MALDI–TOF/TOF and MALDI–Orbitrap platforms. These instruments enable a better resolution of overlapping peaks, structural elucidation of antibiotic degradation products, and the identification of resistance enzymes such as β-lactamases. Their use is particularly beneficial in polymicrobial infections where complex spectral profiles can compromise conventional identification [201]. Furthermore, liquid chromatography (LC)–coupled MALDI (LC-MALDI) workflows allow prior separation of complex protein mixtures, reducing matrix suppression effects and increasing the depth of analysis. This technique has proven useful in proteomics-driven resistance profiling, especially for low-expressed resistance factors in Gram-negative bacteria [212].

The adoption of ML algorithms, including convolutional neural networks and support vector machines, has also elevated AMR detection capabilities. These models can recognize subtle spectral differences and classify resistant versus susceptible isolates with high precision. Recent work by López-Cortés et al. [157] showed over 90% accuracy in detecting carbapenem resistance in *Klebsiella pneumoniae* using ML-enhanced MALDI–TOF MS analysis. Another rapidly evolving innovation is the microdroplet growth assay directly on the MALDI target plate. This phenotypic method monitors bacterial growth in the presence of antibiotics in a matter of hours and has been validated for rapid resistance detection against β-lactams and colistin. Idelevich et al. [74] demonstrated this assay’s clinical value in detecting methicillin resistance in Staphylococcus aureus with high concordance to standard AST methods. Collectively, these technical refinements expand the diagnostic potential of MALDI–TOF MS beyond identification, enabling real-time, high-resolution, and accurate resistance profiling. The ongoing integration of advanced analytical techniques and AI-based models positions MALDI–TOF MS as a cornerstone technology for next-generation AMR surveillance.

## 4. Challenges and Limitations

MALDI–TOF MS has transformed microbial diagnostics by offering rapid and accurate species-level identification. Despite its growing role in clinical and research settings, several challenges limit its broader utility, especially in AMR detection and implementation across diverse healthcare systems. A major limitation lies in the method’s dependence on commercial reference databases. Systems such as the Bruker Biotyper and VITEK MS rely on curated libraries of spectral fingerprints; however, these databases do not always encompass rare, newly emerging, or regionally specific pathogens. This leads to misidentification, particularly among closely related species such as *E. coli* and *Shigella* spp., or within the *Enterobacter cloacae* complex [201,213]. While periodic database updates can address part of this problem, coverage gaps remain a critical obstacle, especially in global surveillance or outbreak scenarios. Another limitation concerns the strain-level resolution of MALDI–TOF MS. Although it reliably distinguishes microorganisms at the species level, it struggles to differentiate between strains—a capacity essential for epidemiological tracking and infection control. For example, the inability to distinguish MRSA from MSSA isolates reduces its value in frontline AMR detection [201]. Research is ongoing to enhance resolution through expanded databases and the integration of ML approaches that extract subtle discriminatory features from complex spectra [11,214].

Sample preparation also influences the quality and reliability of MALDI–TOF MS results. The technique typically requires isolated colonies, meaning that direct analysis of polymicrobial or low-bacterial-load samples—such as blood, cerebrospinal fluid, or tissue biopsies—remains difficult without prior culture enrichment [215]. Moreover, organisms with thick cell walls or lipid-rich membranes, such as Gram-positive bacteria, mycobacteria, and fungi, may require additional steps such as mechanical lysis or chemical extraction to obtain reproducible spectra [213]. These additional requirements limit throughput and reduce the method’s suitability for real-time diagnostics in some clinical scenarios. Cost and accessibility present further barriers, particularly in low- and middle-income countries. Although MALDI–TOF MS is cost-effective in the long term—due to low per-sample costs—its high initial investment, infrastructure requirements, and need for trained personnel limit adoption in under resourced settings. Maintenance costs and the dependence on proprietary reagents further increase the financial burden on laboratories with limited funding [44,201].

Taken together, while MALDI–TOF MS remains a powerful tool for microbial identification and an emerging platform for AMR profiling, its full potential is constrained by technological, logistical, and financial challenges. Overcoming these limitations will require continuous database expansion, improved strain-level discrimination, standardized sample processing protocols, and strategic investments to increase global accessibility. Integration with ML and other omics technologies may help bridge current gaps and extend the utility of MALDI–TOF MS in both clinical microbiology and public health surveillance.

## 5. Future Directions

The evolution of MALDI–TOF MS is poised to expand its diagnostic capabilities while addressing current limitations. A primary objective is the enhancement of reference spectral databases to encompass a broader spectrum of microorganisms, including rare clinical isolates, environmental strains, and geographically specific pathogens. Such expansion is crucial for improving taxonomic resolution, particularly at the strain level, which is essential for precise epidemiological surveillance and outbreak investigations. Artificial intelligence (AI) and ML are anticipated to significantly augment the interpretation of MALDI–TOF MS data. These technologies can uncover complex patterns associated with antimicrobial resistance, enabling earlier and more accurate resistance predictions directly from spectral profiles. The integration of federated learning and cloud-based platforms may further facilitate global data sharing while maintaining data privacy, supporting real-time monitoring of resistance trends.

Hybrid systems that combine MALDI–TOF MS with high-resolution mass analyzers or genomic platforms present a promising avenue for detecting low-abundance biomarkers and differentiating closely related organisms. Additionally, simplifying sample preparation protocols and reducing equipment costs are critical steps to increase accessibility, particularly in resource-limited settings. Ultimately, cross-disciplinary collaboration, regulatory standardization, and continued technological innovation will be necessary to maximize the diagnostic reach of MALDI–TOF MS and embed it within broader public health infrastructures.

## 6. Conclusions

MALDI–TOF MS has transformed modern microbiology by delivering the rapid, reliable, and high-resolution identification of a wide array of pathogens. Its integration into clinical workflows and its expanding utility across food safety, environmental health, and defense sectors underscore its broad impact on public health and disease surveillance. Recent innovations in sample processing, data interpretation, and hybrid instrumentation have elevated the performance of MALDI–TOF MS, especially in the detection of AMR. No longer limited to species-level identification, the technology now facilitates the recognition of resistance-associated phenotypes and molecular markers, aided by ML algorithms and refined spectral databases. These capabilities offer new dimensions in real-time diagnostics and AMR monitoring. Despite these advancements, challenges persist. Variability in inter-laboratory standards, incomplete spectral libraries, and restricted access in resource-limited regions limit the full realization of its global potential. Bridging these gaps requires coordinated efforts in standardization, training, and investment in infrastructure. As diagnostic needs grow more urgent—driven by emerging pathogens and the escalating threat of AMR—MALDI–TOF MS stands at the forefront of microbiological innovation. With continued refinement and equitable implementation, this technology holds the promise to become a global standard in pathogen detection and resistance profiling, guiding faster, smarter, and more targeted infectious disease management.

## Figures and Tables

**Table 2:** Opportunities and considerations for ML-enhanced AMR detection via MALDI–TOF MS.

Category	Details	Key References
Strengths	– Enables high-throughput analysis of complex spectral data	[157]
	– Improves detection of resistance-related features	[159]
	– Reduces reliance on expert interpretation	*
Current Limitations	– Models often lack validation using independent clinical datasets	[157]
	– May not detect novel or evolving resistance mechanisms	[159]
	– Susceptible to false predictions in underrepresented classes	*
Clinical Implications	– Requires robust external validation before routine use	[157]
	– Phenotypic confirmation remains essential for patient safety	[159]
	– Limited utility in settings with rare or emerging pathogens	*
Future Directions	– Integration with larger, diverse datasets to improve model generalizability	*
	– Development of standardized evaluation protocols	[157]
	– Inclusion in diagnostic workflows pending regulatory oversight	[159]

## Data Availability

No new data were created or analyzed in this study. Data sharing is not applicable to this article.

## References

[1] Wat J.K.-H., Xu M., Nan L., Lin H., To K.K.-W., Shum H.C., Hassan S.U. (2025). Rapid antimicrobial susceptibility tests performed by self-diluting microfluidic chips for drug resistance studies and point-of-care diagnostics. Microsyst. Nanoeng..

[2] Mwaturura T., Olaru I.D., Chimhini G., Bwakura-Dangarembizi M., Mangiza M., Chimhuya S., Sado B., Katunga J., Tarupiwa A., Juru A. (2025). Rapid bacterial identification and resistance detection using a low complexity molecular diagnostic platform in Zimbabwe. PLOS Glob. Public Health.

[3] Church D.L., Cerutti L., Gürtler A., Griener T., Zelazny A., Emler S. (2020). Performance and application of 16S rRNA gene cycle sequencing for routine identification of bacteria in the clinical microbiology laboratory. Clin. Microbiol. Rev..

[4] Hassall J., Coxon C., Patel V.C., Goldenberg S.D., Sergaki C. (2024). Limitations of current techniques in clinical antimicrobial resistance diagnosis: Examples and future prospects. NPJ Antimicrob. Resist..

[5] Rossen J., Friedrich A., Moran-Gilad J. (2018). ESCMID Study Group for Genomic and Molecular Diagnostics (ESGMD). Practical issues in implementing whole-genome-sequencing in routine diagnostic microbiology. Clin. Microbiol. Infect..

[6] Robert M.-G., Cornet M., Hennebique A., Rasamoelina T., Caspar Y., Pondérand L., Bidart M., Durand H., Jacquet M., Garnaud C. (2021). MALDI–TOF MS in a medical mycology laboratory: On stage and backstage. Microorganisms.

[7] Barker K.R., Kus J.V., Normand A.-C., Gharabaghi F., McTaggart L., Rotstein C., Richardson S.E., Campigotto A., Tadros M. (2022). A practical workflow for the identification of Aspergillus, Fusarium, Mucorales by MALDI–TOF MS: Database, medium, and incubation optimization. J. Clin. Microbiol..

[8] Park S., Kim D., Ryoo N. (2025). Comparative Assessment of Rapid Identification and Antimicrobial Susceptibility Testing Methods for Bloodstream Infections in a Non-24/7 Clinical Microbiology Laboratory. Microorganisms.

[9] Tsuchida S., Umemura H., Nakayama T. (2020). Current status of matrix-assisted laser desorption/ionization–time-of-flight mass spectrometry (MALDI–TOF MS) in clinical diagnostic microbiology. Molecules.

[10] Chen X.-F., Hou X., Xiao M., Zhang L., Cheng J.-W., Zhou M.-L., Huang J.-J., Zhang J.-J., Xu Y.-C., Hsueh P.-R. (2021). Matrix-assisted laser desorption/ionization time of flight mass spectrometry (MALDI–TOF MS) analysis for the identification of pathogenic microorganisms: A review. Microorganisms.

[11] Singhal N., Kumar M., Kanaujia P.K., Virdi J.S. (2015). MALDI–TOF mass spectrometry: An emerging technology for microbial identification and diagnosis. Front. Microbiol..

[12] Elbehiry A., Aldubaib M., Abalkhail A., Marzouk E., ALbeloushi A., Moussa I., Ibrahem M., Albazie H., Alqarni A., Anagreyyah S. (2022). How MALDI–TOF mass spectrometry technology contributes to microbial infection control in healthcare settings. Vaccines.

[13] Cain T.C., Lubman D.M., Weber W.J. (1994). Differentiation of bacteria using protein profiles from matrix-assisted laser desorption/ionization time-of-flight mass spectrometry. Rapid Commun. Mass. Spectrom..

[14] Kassim A., Pflüger V., Premji Z., Daubenberger C., Revathi G. (2017). Comparison of biomarker based Matrix Assisted Laser Desorption Ionization-Time of Flight Mass Spectrometry (MALDI–TOF MS) and conventional methods in the identification of clinically relevant bacteria and yeast. BMC Microbiol..

[15] Clark A.E., Kaleta E.J., Arora A., Wolk D.M. (2013). Matrix-assisted laser desorption ionization–time of flight mass spectrometry: A fundamental shift in the routine practice of clinical microbiology. Clin. Microbiol. Rev..

[16] Calderaro A., Chezzi C. (2024). MALDI–TOF MS: A reliable tool in the real life of the clinical microbiology laboratory. Microorganisms.

[17] Carbonnelle E., Raskine L. (2011). MALDI–TOF mass spectrometry tools for bacterial identification in clinical microbiology laboratory. Bio. Trib. Mag..

[18] Angeletti S. (2017). Matrix assisted laser desorption time of flight mass spectrometry (MALDI–TOF MS) in clinical microbiology. J. Microbiol. Methods.

[19] Cuénod A., Aerni M., Bagutti C., Bayraktar B., Boz E.S., Carneiro C.B., Casanova C., Coste A.T., Damborg P., van Dam D.W. (2023). Quality of MALDI–TOF mass spectra in routine diagnostics: Results from an international external quality assessment including 36 laboratories from 12 countries using 47 challenging bacterial strains. Clin. Microbiol. Infect..

[20] Welker M., Van Belkum A., Girard V., Charrier J.-P., Pincus D. (2019). An update on the routine application of MALDI–TOF MS in clinical microbiology. Expert. Rev. Proteom..

[21] El Khechine A., Couderc C., Flaudrops C., Raoult D., Drancourt M. (2011). Matrix-assisted laser desorption/ionization time-of-flight mass spectrometry identification of mycobacteria in routine clinical practice. PLoS ONE.

[22] Elbehiry A., Marzouk E., Hamada M., Al-Dubaib M., Alyamani E., Moussa I.M., AlRowaidhan A., Hemeg H.A. (2017). Application of MALDI–TOF MS fingerprinting as a quick tool for identification and clustering of foodborne pathogens isolated from food products. New Microbiol..

[23] Sjöholm M.I., Dillner J., Carlson J. (2008). Multiplex detection of human herpesviruses from archival specimens by using matrix-assisted laser desorption ionization-time of flight mass spectrometry. J. Clin. Microbiol..

[24] Yssouf A., Almeras L., Raoult D., Parola P. (2016). Emerging tools for identification of arthropod vectors. Future Microbiol..

[25] Vega-Rúa A., Pagès N., Fontaine A., Nuccio C., Hery L., Goindin D., Gustave J., Almeras L. (2018). Improvement of mosquito identification by MALDI–TOF MS biotyping using protein signatures from two body parts. Parasites Vectors.

[26] Feucherolles M., Poppert S., Utzinger J., Becker S.L. (2019). MALDI–TOF mass spectrometry as a diagnostic tool in human and veterinary helminthology: A systematic review. Parasites Vectors.

[27] Croxatto A., Prod’hom G., Greub G. (2012). Applications of MALDI–TOF mass spectrometry in clinical diagnostic microbiology. FEMS Microbiol. Rev..

[28] Hou T.-Y., Chiang-Ni C., Teng S.-H. (2019). Current status of MALDI–TOF mass spectrometry in clinical microbiology. J. Food Drug Anal..

[29] Sánchez-Juanes F., Calvo Sánchez N., Belhassen García M., Vieira Lista C., Román R.M., Álamo Sanz R., Muro Álvarez A., Muñoz Bellido J.L. (2022). Applications of MALDI–TOF mass spectrometry to the identification of parasites and arthropod vectors of human diseases. Microorganisms.

[30] Sy I., Conrad L., Becker S.L. (2022). Recent advances and potential future applications of MALDI–TOF mass spectrometry for identification of helminths. Diagnostics.

[31] Do T., Guran R., Adam V., Zitka O. (2022). Use of MALDI–TOF mass spectrometry for virus identification: A review. Analyst.

[32] Otto M. (2009). Staphylococcus epidermidis—the’accidental’pathogen. Nat. Rev. Microbiol..

[33] Banerjee R., Humphries R. (2021). Rapid antimicrobial susceptibility testing methods for blood cultures and their clinical impact. Front. Med..

[34] McLain J.E., Cytryn E., Durso L.M., Young S. (2016). Culture-based methods for detection of antibiotic resistance in agroecosystems: Advantages, challenges, and gaps in knowledge. J. Environ. Qual..

[35] Banerjee R., Teng C.B., Cunningham S.A., Ihde S.M., Steckelberg J.M., Moriarty J.P., Shah N.D., Mandrekar J.N., Patel R. (2015). Randomized trial of rapid multiplex polymerase chain reaction–based blood culture identification and susceptibility testing. Clin. Infect. Dis..

[36] Kommedal Ø., Aasen J.L., Lindemann P.C. (2016). Genetic antimicrobial susceptibility testing in Gram-negative sepsis–impact on time to results in a routine laboratory. APMIS.

[37] Weis C., Cuénod A., Rieck B., Dubuis O., Graf S., Lang C., Oberle M., Brackmann M., Søgaard K.K., Osthoff M. (2022). Direct antimicrobial resistance prediction from clinical MALDI–TOF mass spectra using machine learning. Nat. Med..

[38] Wang H.-Y., Hsieh T.-T., Chung C.-R., Chang H.-C., Horng J.-T., Lu J.-J., Huang J.-H. (2022). Efficiently predicting vancomycin resistance of Enterococcus faecium from MALDI–TOF MS spectra using a deep learning-based approach. Front. Microbiol..

[39] Dhiman N., Hall L., Wohlfiel S.L., Buckwalter S.P., Wengenack N.L. (2011). Performance and cost analysis of matrix-assisted laser desorption ionization–time of flight mass spectrometry for routine identification of yeast. J. Clin. Microbiol..

[40] Fenn J.B., Mann M., Meng C.K., Wong S.F., Whitehouse C.M. (1989). Electrospray ionization for mass spectrometry of large biomolecules. Science.

[41] Anhalt J.P., Fenselau C. (1975). Identification of bacteria using mass spectrometry. Anal. Chem..

[42] Hillenkamp F., Karas M. (1990). Mass spectrometry of peptides and proteins by matrix-assisted ultraviolet laser desorption/ionization. Methods Enzymol..

[43] Fenselau C., Demirev P.A. (2001). Characterization of intact microorganisms by MALDI mass spectrometry. Mass. Spectrom. Rev..

[44] Seng P., Drancourt M., Gouriet F., La Scola B., Fournier P.-E., Rolain J.M., Raoult D. (2009). Ongoing revolution in bacteriology: Routine identification of bacteria by matrix-assisted laser desorption ionization time-of-flight mass spectrometry. Clin. Infect. Dis..

[45] van Belkum A., Chatellier S., Girard V., Pincus D., Deol P., Dunne W.M. (2015). Progress in proteomics for clinical microbiology: MALDI–TOF MS for microbial species identification and more. Expert. Rev. Proteom..

[46] Sauer S., Kliem M. (2010). Mass spectrometry tools for the classification and identification of bacteria. Nat. Rev. Microbiol..

[47] Jarrold M.F. (2021). Applications of charge detection mass spectrometry in molecular biology and biotechnology. Chem. Rev..

[48] Kang J.-S. (2012). Principles and applications of LC-MS/MS for the quantitative bioanalysis of analytes in various biological samples. Tandem Mass Spectrometry—Applications and Principles.

[49] Wieser A., Schneider L., Jung J., Schubert S. (2012). MALDI–TOF MS in microbiological diagnostics—Identification of microorganisms and beyond (mini review). Appl. Microbiol. Biotechnol..

[50] Janiszewska D., Szultka-Młyńska M., Pomastowski P., Buszewski B. (2022). “Omic” approaches to bacteria and antibiotic resistance identification. Int. J. Mol. Sci..

[51] Hsieh S.-Y., Tseng C.-L., Lee Y.-S., Kuo A.-J., Sun C.-F., Lin Y.-H., Chen J.-K. (2008). Highly efficient classification and identification of human pathogenic bacteria by MALDI–TOF MS. Mol. Cell. Proteom..

[52] Marklein G., Josten M., Klanke U., Muller E., Horré R., Maier T., Wenzel T., Kostrzewa M., Bierbaum G., Hoerauf A. (2009). Matrix-assisted laser desorption ionization-time of flight mass spectrometry for fast and reliable identification of clinical yeast isolates. J. Clin. Microbiol..

[53] Sharma M., Gautam V., Mahajan M., Rana S., Majumdar M., Ray P. (2017). Direct identification by matrix-assisted laser desorption ionization-time of flight mass spectrometry (MALDI–TOF MS) from positive blood culture bottles: An opportunity to customize growth conditions for fastidious organisms causing bloodstream infections. Indian. J. Med. Res..

[54] Chen J.H., Ho P.-L., Kwan G.S., She K.K., Siu G.K., Cheng V.C., Yuen K.-Y., Yam W.-C. (2013). Direct bacterial identification in positive blood cultures by use of two commercial matrix-assisted laser desorption ionization–time of flight mass spectrometry systems. J. Clin. Microbiol..

[55] Meex C., Neuville F., Descy J., Huynen P., Hayette M.-P., De Mol P., Melin P. (2012). Direct identification of bacteria from BacT/ALERT anaerobic positive blood cultures by MALDI–TOF MS: MALDI Sepsityper kit versus an in-house saponin method for bacterial extraction. J. Med. Microbiol..

[56] Arroyo M.A., Denys G.A. (2017). Parallel evaluation of the MALDI Sepsityper and Verigene BC-GN assays for rapid identification of Gram-negative bacilli from positive blood cultures. J. Clin. Microbiol..

[57] Conway G.C., Smole S.C., Sarracino D.A., Arbeit R.D., Leopold P.E. (2001). Phyloproteomics: Species identification of Enterobacteriaceae using matrix-assisted laser desorption/ionization time-of-flight mass spectrometry. J. Mol. Microbiol. Biotechnol..

[58] Ferreira L., Sánchez-Juanes F., González-Ávila M., Cembrero-Fuciños D., Herrero-Hernández A., González-Buitrago J.M., Muñoz-Bellido J.L. (2010). Direct identification of urinary tract pathogens from urine samples by matrix-assisted laser desorption ionization-time of flight mass spectrometry. J. Clin. Microbiol..

[59] Segawa S., Sawai S., Murata S., Nishimura M., Beppu M., Sogawa K., Watanabe M., Satoh M., Matsutani T., Kobayashi M. (2014). Direct application of MALDI–TOF mass spectrometry to cerebrospinal fluid for rapid pathogen identification in a patient with bacterial meningitis. Clin. Chim. Acta.

[60] Oviaño M., Bou G. (2018). Matrix-assisted laser desorption ionization–time of flight mass spectrometry for the rapid detection of antimicrobial resistance mechanisms and beyond. Clin. Microbiol. Rev..

[61] Arca-Suárez J., Galán-Sánchez F., Marin-Casanova P., Rodríguez-Iglesias M.A. (2017). Direct identification of microorganisms from thioglycolate broth by MALDI–TOF MS. PLoS ONE.

[62] Wilen C.B., McMullen A.R., Burnham C.-A.D. (2015). Comparison of sample preparation methods, instrumentation platforms, and contemporary commercial databases for identification of clinically relevant mycobacteria by matrix-assisted laser desorption ionization–time of flight mass spectrometry. J. Clin. Microbiol..

[63] Chalupová J., Raus M., Sedlářová M., Šebela M. (2014). Identification of fungal microorganisms by MALDI–TOF mass spectrometry. Biotechnol. Adv..

[64] Cassagne C., Ranque S., Normand A.-C., Fourquet P., Thiebault S., Planard C., Hendrickx M., Piarroux R. (2011). Mould routine identification in the clinical laboratory by matrix-assisted laser desorption ionization time-of-flight mass spectrometry. PLoS ONE.

[65] Jazii F.R., Najafi Z., Malekzadeh R., Conrads T.P., Ziaee A.A., Abnet C., Yazdznbod M., Karkhane A.A., Salekdeh G.H. (2006). Identification of squamous cell carcinoma associated proteins by proteomics and loss of beta tropomyosin expression in esophageal cancer. World J. Gastroenterol. WJG.

[66] Connolly J.P., Comerci D., Alefantis T.G., Walz A., Quan M., Chafin R., Grewal P., Mujer C.V., Ugalde R.A., DelVecchio V.G. (2006). Proteomic analysis of Brucella abortus cell envelope and identification of immunogenic candidate proteins for vaccine development. Proteomics.

[67] Qi Y.J., He Q.Y., Ma Y.F., Du Y.W., Liu G.C., Li Y.J., Tsao G.S., Ngai S.M., Chiu J.F. (2008). Proteomic identification of malignant transformation-related proteins in esophageal squamous cell carcinoma. J. Cell. Biochem..

[68] Gagnaire J., Dauwalder O., Boisset S., Khau D., Freydiere A.-M., Ader F., Bes M., Lina G., Tristan A., Reverdy M.-E. (2012). Detection of Staphylococcus aureus delta-toxin production by whole-cell MALDI–TOF mass spectrometry. PLoS ONE.

[69] Alam S.I., Kumar B., Kamboj D.V. (2012). Multiplex detection of protein toxins using MALDI–TOF-TOF tandem mass spectrometry: Application in unambiguous toxin detection from bioaerosol. Anal. Chem..

[70] Cassagne C., Normand A.C., L’Ollivier C., Ranque S., Piarroux R. (2016). Performance of MALDI-TOF MS platforms for fungal identification. Mycoses.

[71] Patel R. (2019). A moldy application of MALDI: MALDI–ToF mass spectrometry for fungal identification. J. Fungi.

[72] Singhal N., Kumar M., Virdi J.S. (2016). MALDI–TOF MS in clinical parasitology: Applications, constraints and prospects. Parasitology.

[73] Huguenin A., Depaquit J., Villena I., Ferté H. (2019). MALDI–TOF mass spectrometry: A new tool for rapid identification of cercariae (Trematoda, Digenea). Parasite.

[74] Idelevich E., Sparbier K., Kostrzewa M., Becker K. (2018). Rapid detection of antibiotic resistance by MALDI–TOF mass spectrometry using a novel direct-on-target microdroplet growth assay. Clin. Microbiol. Infect..

[75] Alizadeh M., Yousefi L., Pakdel F., Ghotaslou R., Rezaee M.A., Khodadadi E., Oskouei M.A., Soroush Barhaghi M.H., Kafil H.S. (2021). MALDI-TOF mass spectroscopy applications in clinical microbiology. Adv. Pharmacol. Pharm. Sci..

[76] Calderaro A., Arcangeletti M.-C., Rodighiero I., Buttrini M., Gorrini C., Motta F., Germini D., Medici M.-C., Chezzi C., De Conto F. (2014). Matrix-assisted laser desorption/ionization time-of-flight (MALDI–TOF) mass spectrometry applied to virus identification. Sci. Rep..

[77] Cobo F. (2013). Application of MALDI–TOF mass spectrometry in clinical virology: A review. Open Virol. J..

[78] Majchrzykiewicz-Koehorst J.A., Heikens E., Trip H., Hulst A.G., de Jong A.L., Viveen M.C., Sedee N.J., van der Plas J., Coenjaerts F.E., Paauw A. (2015). Rapid and generic identification of influenza A and other respiratory viruses with mass spectrometry. J. Virol. Methods.

[79] Bader O. (2013). MALDI-TOF-MS-based species identification and typing approaches in medical mycology. Proteomics.

[80] Vella A., De Carolis E., Mello E., Perlin D.S., Sanglard D., Sanguinetti M., Posteraro B. (2017). Potential use of MALDI–ToF mass spectrometry for rapid detection of antifungal resistance in the human pathogen Candida glabrata. Sci. Rep..

[81] Culha G., Akyar I., Zeyrek F.Y., Kurt Ö., Gündüz C., Töz S.Ö., Östan I., Cavus I., Gülkan B., Kocagöz T. (2014). Leishmaniasis in Turkey: Determination of Leishmania species by matrix-assisted laser desorption ionization time-of-flight mass spectrometry (MALDI–TOF MS). Iran. J. Parasitol..

[82] Magnuson M.L., Owens J.H., Kelty C.A. (2000). Characterization of Cryptosporidium parvum by matrix-assisted laser desorption ionization–time of flight mass spectrometry. Appl. Environ. Microbiol..

[83] Kim J., Bae S.-S., Sung M.-H., Lee K.-H., Park S.-J. (2009). Comparative proteomic analysis of trophozoites versus cysts of Giardia lamblia. Parasitol. Res..

[84] Calderaro A., Piergianni M., Buttrini M., Montecchini S., Piccolo G., Gorrini C., Rossi S., Chezzi C., Arcangeletti M.C., Medici M.C. (2015). MALDI–TOF mass spectrometry for the detection and differentiation of Entamoeba histolytica and Entamoeba dispar. PLoS ONE.

[85] He Y., Li H., Lu X., Stratton C.W., Tang Y.-W. (2010). Mass spectrometry biotyper system identifies enteric bacterial pathogens directly from colonies grown on selective stool culture media. J. Clin. Microbiol..

[86] Ruiz-Aragon J., Ballestero-Tellez M., Gutierrez-Gutierrez B., de Cueto M., Rodriguez-Bano J., Pascual A. (2018). Direct bacterial identification from positive blood cultures using matrix-assisted laser desorption/ionization time-of-flight (MALDI–TOF) mass spectrometry: A systematic review and meta-analysis. Enfermedades Infecc. Microbiol. Clínica.

[87] Axelsson C., Rehnstam-Holm A.-S., Nilson B. (2020). Rapid detection of antibiotic resistance in positive blood cultures by MALDI–TOF MS and an automated and optimized MBT-ASTRA protocol for Escherichia coli and Klebsiella pneumoniae. Infect. Dis..

[88] Verroken A., Defourny L., Lechgar L., Magnette A., Delmée M., Glupczynski Y. (2015). Reducing time to identification of positive blood cultures with MALDI–TOF MS analysis after a 5-h subculture. Eur. J. Clin. Microbiol. Infect. Dis..

[89] Clerc O., Prod’hom G., Vogne C., Bizzini A., Calandra T., Greub G. (2013). Impact of matrix-assisted laser desorption ionization time-of-flight mass spectrometry on the clinical management of patients with Gram-negative bacteremia: A prospective observational study. Clin. Infect. Dis..

[90] Li C., Ding S., Huang Y., Wang Z., Shen J., Ling H., Xu Y. (2018). Detection of AmpC β-lactamase-producing Gram-negative bacteria by matrix-assisted laser desorption/ionization time-of-flight mass spectrometry. J. Hosp. Infect..

[91] Li D., Yi J., Han G., Qiao L. (2022). MALDI–TOF mass spectrometry in clinical analysis and research. ACS Meas. Sci. Au.

[92] Wang K., Shu C., Soberón M., Bravo A., Zhang J. (2018). Systematic characterization of Bacillus genetic stock center Bacillus thuringiensis strains using multi-locus sequence typing. J. Invertebr. Pathol..

[93] Claydon M.A., Davey S.N., Edwards-Jones V., Gordon D.B. (1996). The rapid identification of intact microorganisms using mass spectrometry. Nat. Biotechnol..

[94] Demirev P.A., Ho Y.-P., Ryzhov V., Fenselau C. (1999). Microorganism identification by mass spectrometry and protein database searches. Anal. Chem..

[95] Hettick J.M., Kashon M.L., Simpson J.P., Siegel P.D., Mazurek G.H., Weissman D.N. (2004). Proteomic profiling of intact mycobacteria by matrix-assisted laser desorption/ionization time-of-flight mass spectrometry. Anal. Chem..

[96] Holland R., Wilkes J., Rafii F., Sutherland J., Persons C., Voorhees K., Lay J. (1996). Rapid identification of intact whole bacteria based on spectral patterns using matrix-assisted laser desorption/ionization with time-of-flight mass spectrometry. Rapid Commun. Mass. Spectrom..

[97] McMullen A.R., Wallace M.A., Pincus D.H., Wilkey K., Burnham C.-A.D. (2016). Evaluation of the Vitek MS matrix-assisted laser desorption ionization–time of flight mass spectrometry system for identification of clinically relevant filamentous fungi. J. Clin. Microbiol..

[98] Won E.J., Shin J.H., Lee K., Kim M.-N., Lee H.S., Park Y.-J., Joo M.Y., Kim S.H., Shin M.G., Suh S.P. (2013). Accuracy of Species-Level Identification of Yeast Isolates from Blood Cultures from 10 University Hospitals in South Korea by Use of the Matrix-Assisted Laser Desorption Ionization–Time of Flight Mass Spectrometry-Based Vitek MS System. J. Clin. Microbiol..

[99] Pence M., McElvania TeKippe E., Wallace M., Burnham C.-A.D. (2014). Comparison and optimization of two MALDI–TOF MS platforms for the identification of medically relevant yeast species. Eur. J. Clin. Microbiol. Infect. Dis..

[100] Hrabák J., Chudáčková E., Walková R. (2013). Matrix-assisted laser desorption ionization–time of flight (MALDI–TOF) mass spectrometry for detection of antibiotic resistance mechanisms: From research to routine diagnosis. Clin. Microbiol. Rev..

[101] Camara J.E., Hays F.A. (2007). Discrimination between wild-type and ampicillin-resistant Escherichia coli by matrix-assisted laser desorption/ionization time-of-flight mass spectrometry. Anal. Bioanal. Chem..

[102] Dubska L., Pilatova K., Dolejska M., Bortlicek Z., Frostova T., Literak I., Valik D. (2011). Surface-enhanced laser desorption ionization/time-of-flight (SELDI-TOF) mass spectrometry (MS) as a phenotypic method for rapid identification of antibiotic resistance. Anaerobe.

[103] Zhu Y., Gasilova N., Jović M., Qiao L., Liu B., Lovey L.T., Pick H., Girault H.H. (2018). Detection of antimicrobial resistance-associated proteins by titanium dioxide-facilitated intact bacteria mass spectrometry. Chem. Sci..

[104] Franz C., Den Besten H.M., Boehnlein C., Gareis M., Zwietering M.H., Fusco V. (2018). Microbial food safety in the 21st century: Emerging challenges and foodborne pathogenic bacteria. Trends Food Sci. Technol..

[105] Barker S.F., Amoah P., Drechsel P. (2014). A probabilistic model of gastroenteritis risks associated with consumption of street food salads in Kumasi, Ghana: Evaluation of methods to estimate pathogen dose from water, produce or food quality. Sci. Total Environ..

[106] Naeem M., Bourassa D. (2024). Optimizing Poultry Nutrition to Combat Salmonella: Insights from the Literature. Microorganisms.

[107] Riley M.F. (2021). One Health Pandemic Prevention and Mitigation. Food Drug Law. J..

[108] Dai G., Yao H., Yang L., Ding Y., Du S., Shen H., Mo F. (2023). Rapid detection of foodborne pathogens in diverse foodstuffs by universal electrochemical aptasensor based on UiO-66 and methylene blue composites. Food Chem..

[109] Rivera D., Toledo V., Reyes-Jara A., Navarrete P., Tamplin M., Kimura B., Wiedmann M., Silva P., Switt A.I.M. (2018). Approaches to empower the implementation of new tools to detect and prevent foodborne pathogens in food processing. Food Microbiol..

[110] Gao R., Liu X., Xiong Z., Wang G., Ai L. (2024). Research progress on detection of foodborne pathogens: The more rapid and accurate answer to food safety. Food Res. Int..

[111] Pavlovic M., Huber I., Konrad R., Busch U. (2013). Application of MALDI–TOF MS for the identification of food borne bacteria. Open Microbiol. J..

[112] Han S., Jeong Y., Choi S. (2021). Current scenario and challenges in the direct identification of microorganisms using MALDI TOF MS. Microorganisms.

[113] Biswas S., Rolain J.-M. (2013). Use of MALDI–TOF mass spectrometry for identification of bacteria that are difficult to culture. J. Microbiol. Methods.

[114] Rychert J. (2019). Benefits and limitations of MALDI–TOF mass spectrometry for the identification of microorganisms. J. Infect. Epidemiol..

[115] Shafini A., Son R., Mahyudin N., Rukayadi Y., Zainazor T.T. (2017). Prevalence of Salmonella spp. in chicken and beef from retail outlets in Malaysia. Int. Food Res. J..

[116] Khater D.F., Lela R.A., El-Diasty M., Moustafa S.A., Wareth G. (2021). Detection of harmful foodborne pathogens in food samples at the points of sale by MALDT-TOF MS in Egypt. BMC Res. Notes.

[117] Xu X., Liu G., Huang X., Li L., Lin H., Xu D. (2021). MALDI–TOF MS-based identification of bacteria and a survey of fresh vegetables with pathogenic bacteria in Beijing, China. Food Biosci..

[118] Cebeci T. (2019). A survey of raw milk for microbiological quality and typing of foodborne pathogens by MALDI–TOF MS. Adnan Menderes Üniversitesi Ziraat Fakültesi Derg..

[119] Sulaiman I.M., Miranda N., Simpson S. (2021). MALDI–TOF mass spectrometry and 16S rRNA gene sequence analysis for the identification of *Foodborne clostridium* spp. J. AOAC Int..

[120] Elbehiry A., Marzouk E., Moussa I.M., Dawoud T.M., Mubarak A.S., Al-Sarar D., Alsubki R.A., Alhaji J.H., Hamada M., Abalkhail A. (2021). Acinetobacter baumannii as a community foodborne pathogen: Peptide mass fingerprinting analysis, genotypic of biofilm formation and phenotypic pattern of antimicrobial resistance. Saudi J. Biol. Sci..

[121] Quéro L., Girard V., Pawtowski A., Tréguer S., Weill A., Arend S., Cellière B., Polsinelli S., Monnin V., van Belkum A. (2019). Development and application of MALDI–TOF MS for identification of food spoilage fungi. Food Microbiol..

[122] Pattabhiramaiah M., Mallikarjunaiah S. (2021). High-Throughput Sequencing for Detection of Foodborne Pathogens in Food Safety. Sequencing Technologies in Microbial Food Safety and Quality.

[123] Hasan N., Zanuddin N. (2018). Molecular identification of isolated fungi from banana, mango and pineapple spoiled fruits. AIP Conference Proceedings.

[124] Quintilla R., Kolecka A., Casaregola S., Daniel H.M., Houbraken J., Kostrzewa M., Boekhout T., Groenewald M. (2018). MALDI–TOF MS as a tool to identify foodborne yeasts and yeast-like fungi. Int. J. Food Microbiol..

[125] Ahmadsah L.S., Kim E., Jung Y.-S., Kim H.-Y. (2018). Identification of LAB and fungi in Laru, a fermentation starter, by PCR-DGGE, SDS-PAGE, and MALDI–TOF MS. J. Microbiol. Biotechnol..

[126] Bader O. (2016). Fungal species identification by MALDI–ToF mass spectrometry. Human Fungal Pathogen Identification: Methods and Protocols.

[127] Dash J., Naykodi A., Mohakud N.K., Deb S. (2024). MALDI TOF-MS for microbial identification and diagnosis. Evolving Landscape of Molecular Diagnostics.

[128] Qi G., Hao L., Gan Y., Xin T., Lou Q., Xu W., Song J. (2024). Identification of closely related species in Aspergillus through Analysis of Whole-Genome. Front. Microbiol..

[129] Clark C.M., Costa M.S., Sanchez L.M., Murphy B.T. (2018). Coupling MALDI–TOF mass spectrometry protein and specialized metabolite analyses to rapidly discriminate bacterial function. Proc. Natl. Acad. Sci. USA.

[130] Seuylemezian A., Aronson H.S., Tan J., Lin M., Schubert W., Vaishampayan P. (2018). Development of a custom MALDI–TOF MS database for species-level identification of bacterial isolates collected from spacecraft and associated surfaces. Front. Microbiol..

[131] Tuohy J.M., Mueller-Spitz S.R., Albert C.M., Scholz-Ng S.E., Wall M.E., Noutsios G.T., Gutierrez A.J., Sandrin T.R. (2018). MALDI–TOF MS affords discrimination of Deinococcus aquaticus isolates obtained from diverse biofilm habitats. Front. Microbiol..

[132] Kurli R., Chaudhari D., Pansare A.N., Khairnar M., Shouche Y.S., Rahi P. (2018). Cultivable microbial diversity associated with cellular phones. Front. Microbiol..

[133] Rahi P., Prakash O., Shouche Y.S. (2016). Matrix-assisted laser desorption/ionization time-of-flight mass-spectrometry (MALDI–TOF MS) based microbial identifications: Challenges and scopes for microbial ecologists. Front. Microbiol..

[134] Jang K.-S., Kim Y.H. (2018). Rapid and robust MALDI–TOF MS techniques for microbial identification: A brief overview of their diverse applications. J. Microbiol..

[135] Santos I.C., Hildenbrand Z.L., Schug K.A. (2016). Applications of MALDI–TOF MS in environmental microbiology. Analyst.

[136] Ashfaq M.Y., Da’na D.A., Al-Ghouti M.A. (2022). Application of MALDI–TOF MS for identification of environmental bacteria: A review. J. Environ. Manag..

[137] Niestępski S., Harnisz M., Korzeniewska E., Osińska A. (2019). Isolation of anaerobic bacteria of the Bacteroides fragilis group from environmental samples. Proc. E3S Web Conf..

[138] Fehlberg L.C.C., Andrade L.H.S., Assis D.M., Pereira R.H.V., Gales A.C., Marques E.A. (2013). Performance of MALDI–ToF MS for species identification of Burkholderia cepacia complex clinical isolates. Diagn. Microbiol. Infect. Dis..

[139] Vicenzi F.J., Pillonetto M., Souza H.A.P.H.d.M.d., Palmeiro J.K., Riedi C.A., Rosario-Filho N.A., Dalla-Costa L.M. (2016). Polyphasic characterisation of Burkholderia cepacia complex species isolated from children with cystic fibrosis. Memórias Inst. Oswaldo Cruz.

[140] Furlan J.P.R., Pitondo-Silva A., Braz V.S., Gallo I.F.L., Stehling E.G. (2019). Evaluation of different molecular and phenotypic methods for identification of environmental Burkholderia cepacia complex. World J. Microbiol. Biotechnol..

[141] Hazen T.H., Martinez R.J., Chen Y., Lafon P.C., Garrett N.M., Parsons M.B., Bopp C.A., Sullards M.C., Sobecky P.A. (2009). Rapid identification of Vibrio parahaemolyticus by whole-cell matrix-assisted laser desorption ionization-time of flight mass spectrometry. Appl. Environ. Microbiol..

[142] Sulaiman I.M., Banerjee P., Hsieh Y.-H., Miranda N., Simpson S., Kerdahi K. (2018). Rapid detection of Staphylococcus aureus and related species isolated from food, environment, cosmetics, a medical device, and clinical samples using the VITEK MS microbial identification system. J. AOAC Int..

[143] Konate S., Camara A., Lo C., Tidjani Alou M., Hamidou Togo A., Niare S., Armstrong N., Djimdé A., Thera M., Fenollar F. (2021). Virgibacillus doumboii sp. nov., a halophilic bacterium isolated from the stool of a healthy child in Mali. New Microbes New Infect.

[144] Brauge T., Trigueros S., Briet A., Debuiche S., Leleu G., Gassilloud B., Wilhelm A., Py J.-S., Midelet G. (2021). MALDI–TOF mass spectrometry fingerprinting performance versus 16S rDNA sequencing to identify bacterial microflora from seafood products and sea water samples. Front. Mar. Sci..

[145] Pinar-Méndez A., Fernández S., Baquero D., Vilaró C., Galofré B., González S., Rodrigo-Torres L., Arahal D.R., Macián M.C., Ruvira M.A. (2021). Rapid and improved identification of drinking water bacteria using the Drinking Water Library, a dedicated MALDI–TOF MS database. Water Res..

[146] Emami K., Askari V., Ullrich M., Mohinudeen K., Anil A.C., Khandeparker L., Burgess J.G., Mesbahi E. (2012). Characterization of bacteria in ballast water using MALDI–TOF mass spectrometry. PLoS ONE.

[147] Fergusson C.H., Coloma J.M., Valentine M.C., Haeckl F.J., Linington R.G. (2020). Custom matrix-assisted laser desorption ionization–time of flight mass spectrometric database for identification of environmental isolates of the genus Burkholderia and related genera. Appl. Environ. Microbiol..

[148] Henderson D.A. (1998). Bioterrorism as a public health threat. Emerg. Infect. Dis..

[149] Shaw E.I., Moura H., Woolfitt A.R., Ospina M., Thompson H.A., Barr J.R. (2004). Identification of Biomarkers of Whole Coxiella b urnetii Phase I by MALDI–TOF Mass Spectrometry. Anal. Chem..

[150] Pierce C.Y., Barr J.R., Woolfitt A.R., Moura H., Shaw E.I., Thompson H.A., Massung R.F., Fernandez F.M. (2007). Strain and phase identification of the US category B agent Coxiella burnetii by matrix assisted laser desorption/ionization time-of-flight mass spectrometry and multivariate pattern recognition. Anal. Chim. Acta.

[151] Lasch P., Beyer W., Nattermann H., Stämmler M., Siegbrecht E., Grunow R., Naumann D. (2009). Identification of Bacillus anthracis by using matrix-assisted laser desorption ionization-time of flight mass spectrometry and artificial neural networks. Appl. Environ. Microbiol..

[152] Seibold E., Maier T., Kostrzewa M., Zeman E., Splettstoesser W. (2010). Identification of Francisella tularensis by whole-cell matrix-assisted laser desorption ionization-time of flight mass spectrometry: Fast, reliable, robust, and cost-effective differentiation on species and subspecies levels. J. Clin. Microbiol..

[153] Scholl P.F., Leonardo M.A., Rule A.M., Carlson M.A., Antoine M.D., Buckley T.J. (1999). The development of matrix-assisted laser desorption/ionization time-of-flight mass spectrometry for the detection of biological warfare agent aerosols. Johns. Hopkins APL Tech. Dig..

[154] Jeong Y.-S., Lee J., Kim S.-J. (2013). Discrimination of Bacillus anthracis spores by direct in-situ analysis of matrix-assisted laser desorption/ionization time-of-flight mass spectrometry. Bull. Korean Chem. Soc..

[155] Jeong Y.S., Choi S., Chong E., Kim J., Kim S.J. (2014). Rapid detection of Bacillus spore aerosol particles by direct in situ analysis using MALDI-TOF mass spectrometry. Lett. Appl. Microbiol..

[156] Sparbier K., Lange C., Jung J., Wieser A., Schubert S., Kostrzewa M. (2013). MALDI biotyper-based rapid resistance detection by stable-isotope labeling. J. Clin. Microbiol..

[157] López-Cortés X.A., Manríquez-Troncoso J.M., Sepúlveda A.Y., Soto P.S. (2025). Integrating Machine Learning with MALDI–TOF Mass Spectrometry for Rapid and Accurate Antimicrobial Resistance Detection in Clinical Pathogens. Int. J. Mol. Sci..

[158] Astudillo C.A., López-Cortés X.A., Ocque E., Manríquez-Troncoso J.M. (2024). Multi-label classification to predict antibiotic resistance from raw clinical MALDI–TOF mass spectrometry data. Sci. Rep..

[159] Ren M., Chen Q., Zhang J. (2024). Repurposing MALDI–TOF MS for effective antibiotic resistance screening in Staphylococcus epidermidis using machine learning. Sci. Rep..

[160] Lin H.-T.V., Yang T.-W., Lu W.-J., Chiang H.-J., Hsu P.-H. (2025). Machine Learning-Enhanced MALDI–TOF MS for Real-Time Detection of Antibiotic-Resistant *E. coli* in Food Processing. LWT.

[161] Reller L.B., Weinstein M., Jorgensen J.H., Ferraro M.J. (2009). Antimicrobial susceptibility testing: A review of general principles and contemporary practices. Clin. Infect. Dis..

[162] Florio W., Morici P., Ghelardi E., Barnini S., Lupetti A. (2018). Recent advances in the microbiological diagnosis of bloodstream infections. Crit. Rev. Microbiol..

[163] van Belkum A., Bachmann T.T., Lüdke G., Lisby J.G., Kahlmeter G., Mohess A., Becker K., Hays J.P., Woodford N., Mitsakakis K. (2019). Developmental roadmap for antimicrobial susceptibility testing systems. Nat. Rev. Microbiol..

[164] Ellington M., Ekelund O., Aarestrup F.M., Canton R., Doumith M., Giske C., Grundman H., Hasman H., Holden M., Hopkins K.L. (2017). The role of whole genome sequencing in antimicrobial susceptibility testing of bacteria: Report from the EUCAST Subcommittee. Clin. Microbiol. Infect..

[165] Weis C., Cuénod A., Rieck B., Llinares-López F., Dubuis O., Graf S., Lang C., Oberle M., Soegaard K.K., Osthoff M. (2020). Direct Antimicrobial RSesistance Prediction from MALDI–TOF mass spectra profile in clinical isolates through Machine Learning. BioRxiv.

[166] Schubert S., Kostrzewa M. (2017). MALDI–TOF MS in the microbiology laboratory: Current trends. Curr. Issues Mol. Biol..

[167] Rodríguez-Sánchez B., Alcalá L., Marín M., Ruiz A., Alonso E., Bouza E. (2016). Evaluation of MALDI–TOF MS (matrix-assisted laser desorption-ionization time-of-flight mass spectrometry) for routine identification of anaerobic bacteria. Anaerobe.

[168] CLSI (2023). Performance Standards For Antimicrobial Susceptibility Testing (33rd ed.). CLSI Supplement M100. https://clsi.org/shop/standards/m100/.

[169] Card R.M., Warburton P.J., MacLaren N., Mullany P., Allan E., Anjum M.F. (2014). Application of microarray and functional-based screening methods for the detection of antimicrobial resistance genes in the microbiomes of healthy humans. PLoS ONE.

[170] De Waele G., Menschaert G., Waegeman W. (2024). An antimicrobial drug recommender system using MALDI–TOF MS and dual-branch neural networks. eLife.

[171] Murray C.J., Ikuta K.S., Sharara F., Swetschinski L., Aguilar G.R., Gray A., Han C., Bisignano C., Rao P., Wool E. (2022). Global burden of bacterial antimicrobial resistance in 2019: A systematic analysis. Lancet.

[172] O’Neill J. (2016). Tackling Drug-Resistant Infections Globally: Final Report and Recommendations. Wellcome Trust and the Department of Health, UK Government. https://amr-review.org/sites/default/files/160518_Final%20paper_with%20cover.pdf.

[173] Khan Z.A., Siddiqui M.F., Park S. (2019). Current and emerging methods of antibiotic susceptibility testing. Diagnostics.

[174] Humphries R.M. (2022). Ad hoc antimicrobial susceptibility testing from MALDI–TOF MS spectra in the clinical microbiology laboratory. Clin. Chem..

[175] Weis C., Horn M., Rieck B., Cuénod A., Egli A., Borgwardt K. (2020). Topological and kernel-based microbial phenotype prediction from MALDI–TOF mass spectra. Bioinformatics.

[176] Shlaes D.M., Gerding D.N., John J.F., Craig W.A., Bornstein D.L., Duncan R.A., Eckman M.R., Farrer W.E., Greene W.H., Lorian V. (1997). Society for Healthcare Epidemiology of America and Infectious Diseases Society of America Joint Committee on the Prevention of Antimicrobial Resistance guidelines for the prevention of antimicrobial resistance in hospitals. Infect. Control Hosp. Epidemiol..

[177] Bai J., Fan Z., Zhang L., Xu X., Zhang Z. Classification of methicillin-resistant and methicillin-susceptible staphylococcus aureus using an improved genetic algorithm for feature selection based on mass spectra. Proceedings of the 9th International Conference on Bioinformatics and Biomedical Technology.

[178] Soo Y.T., Waled S.N.M.B., Ng S., Peh Y.H., Chew K.L. (2020). Evaluation of EUCAST rapid antimicrobial susceptibility testing (RAST) directly from blood culture bottles. Eur. J. Clin. Microbiol. Infect. Dis..

[179] Webber D.M., Wallace M.A., Burnham C.-A.D. (2022). Stop waiting for tomorrow: Disk diffusion performed on early growth is an accurate method for antimicrobial susceptibility testing with reduced turnaround time. J. Clin. Microbiol..

[180] van den Bijllaardt W., Buiting A.G., Mouton J.W., Muller A.E. (2017). Shortening the incubation time for antimicrobial susceptibility testing by disk diffusion for Enterobacteriaceae: How short can it be and are the results accurate?. Int. J. Antimicrob. Agents.

[181] Lange C., Schubert S., Jung J., Kostrzewa M., Sparbier K. (2014). Quantitative matrix-assisted laser desorption ionization–time of flight mass spectrometry for rapid resistance detection. J. Clin. Microbiol..

[182] Wilhelm C.M., Carneiro M.d.S., Inamine E., Barth A.L. (2023). A rapid and easy method of MALDI biotyper antibiotic susceptibility test rapid assay to provide early meropenem susceptibility profile in enterobacterales. Microbiol. Spectr..

[183] Gibb S., Strimmer K. (2012). MALDIquant: A versatile R package for the analysis of mass spectrometry data. Bioinformatics.

[184] Yoon E.-J., Jeong S.H. (2021). MALDI–TOF mass spectrometry technology as a tool for the rapid diagnosis of antimicrobial resistance in bacteria. Antibiotics.

[185] Vrioni G., Tsiamis C., Oikonomidis G., Theodoridou K., Kapsimali V., Tsakris A. (2018). MALDI–TOF mass spectrometry technology for detecting biomarkers of antimicrobial resistance: Current achievements and future perspectives. Ann. Transl. Med..

[186] Ramzan M., Raza A., un Nisa Z., Abdel-Massih R.M., Al Bakain R., Cabrerizo F.M., Cruz T.E.D., Aziz R.K., Musharraf S.G. (2024). Detection of antimicrobial resistance (AMR) and antimicrobial susceptibility testing (AST) using advanced spectroscopic techniques: A review. TrAC Trends Anal. Chem..

[187] Viboud G., Asaro H., Huang M.B. (2024). Use of matrix-assisted laser desorption ionization time of flight (MALDI–TOF) to detect antibiotic resistance in bacteria: A scoping review. Am. J. Clin. Pathol..

[188] Li J., Huang Y., Hu Y., Sun Q., Cai J., Zhou H., Gu D., Chen G., Wang Y., Zhang R. (2022). A rapid MALDI-TOF mass spectrometry-based method for colistin susceptibility testing in Escherichia coli. Microb. Biotechnol..

[189] Wang G., Song G., Xu Y. (2021). A rapid antimicrobial susceptibility test for Klebsiella pneumoniae using a broth micro-dilution combined with MALDI TOF MS. Infect. Drug Resist..

[190] Sparbier K., Schubert S., Kostrzewa M. (2016). MBT-ASTRA: A suitable tool for fast antibiotic susceptibility testing?. Methods.

[191] Ceyssens P.-J., Soetaert K., Timke M., Van den Bossche A., Sparbier K., De Cremer K., Kostrzewa M., Hendrickx M., Mathys V. (2017). Matrix-assisted laser desorption ionization–time of flight mass spectrometry for combined species identification and drug sensitivity testing in mycobacteria. J. Clin. Microbiol..

[192] Furniss R.C.D., Dortet L., Bolland W., Drews O., Sparbier K., Bonnin R.A., Filloux A., Kostrzewa M., Mavridou D.A., Larrouy-Maumus G. (2019). Detection of colistin resistance in Escherichia coli by use of the MALDI Biotyper Sirius mass spectrometry system. J. Clin. Microbiol..

[193] Li Y., Li D., Huang X., Long S., Yu H., Zhang J. (2024). Temporal Shifts in Etiological Agents and Antibiotic Resistance Patterns of Biliary Tract Infections in Sichuan Province, China (2017–2023). Infect. Drug Resist..

[194] Du Z., Yang R., Guo Z., Song Y., Wang J. (2002). Identification of Staphylococcus a ureus and determination of its methicillin resistance by matrix-assisted laser desorption/ionization time-of-flight mass spectrometry. Anal. Chem..

[195] Josten M., Dischinger J., Szekat C., Reif M., Al-Sabti N., Sahl H.-G., Parcina M., Bekeredjian-Ding I., Bierbaum G. (2014). Identification of agr-positive methicillin-resistant Staphylococcus aureus harbouring the class A mec complex by MALDI–TOF mass spectrometry. Int. J. Med. Microbiol..

[196] Lau A.F., Wang H., Weingarten R.A., Drake S.K., Suffredini A.F., Garfield M.K., Chen Y., Gucek M., Youn J.-H., Stock F. (2014). A rapid matrix-assisted laser desorption ionization–time of flight mass spectrometry-based method for single-plasmid tracking in an outbreak of carbapenem-resistant Enterobacteriaceae. J. Clin. Microbiol..

[197] Cordovana M., Kostrzewa M., Glandorf J., Bienia M., Ambretti S., Pranada A.B. (2018). A full MALDI-based approach to detect plasmid-encoded KPC-producing Klebsiella pneumoniae. Front. Microbiol..

[198] Figueroa-Espinosa R., Costa A., Cejas D., Barrios R., Vay C., Radice M., Gutkind G., Di Conza J. (2019). MALDI–TOF MS based procedure to detect KPC-2 directly from positive blood culture bottles and colonies. J. Microbiol. Methods.

[199] Cordovana M., Pranada A.B., Ambretti S., Kostrzewa M. (2019). MALDI–TOF bacterial subtyping to detect antibiotic resistance. Clin. Mass. Spectrom..

[200] Chang K.C., Chung C.Y., Yeh C.H., Hsu K.H., Chin Y.C., Huang S.S., Liu B.R., Chen H.A., Hu A., Soo P.C. (2018). Direct detection of carbapenemase-associated proteins of Acinetobacter baumannii using nanodiamonds coupled with matrix-assisted laser desorption/ionization time-of-flight mass spectrometry. J. Microbiol. Methods.

[201] Florio W., Baldeschi L., Rizzato C., Tavanti A., Ghelardi E., Lupetti A. (2020). Detection of antibiotic-resistance by MALDI–TOF mass spectrometry: An expanding area. Front. Cell. Infect. Microbiol..

[202] Flores-Treviño S., Garza-González E., Mendoza-Olazarán S., Morfín-Otero R., Camacho-Ortiz A., Rodríguez-Noriega E., Martínez-Meléndez A., Bocanegra-Ibarias P. (2019). Screening of biomarkers of drug resistance or virulence in ESCAPE pathogens by MALDI–TOF mass spectrometry. Sci. Rep..

[203] Feucherolles M. (2024). Integrating MALDI–TOF mass spectrometry with machine learning techniques for rapid antimicrobial resistance screening of foodborne bacterial pathogens. Foodborne Bacterial Pathogens: Methods and Protocols.

[204] Cai H., Xing X., Su Y., Yang C. (2025). Innovative applications and future perspectives of chromatography-mass spectrometry in drug research. Front. Pharmacol..

[205] Nguyen D.C., Pham Q.-V., Pathirana P.N., Ding M., Seneviratne A., Lin Z., Dobre O., Hwang W.-J. (2022). Federated learning for smart healthcare: A survey. ACM Comput. Surv..

[206] Prayitno, Shyu C.-R., Putra K.T., Chen H.-C., Tsai Y.-Y., Hossain K.T., Jiang W., Shae Z.-Y. (2021). A systematic review of federated learning in the healthcare area: From the perspective of data properties and applications. Appl. Sci..

[207] Li M., Xu P., Hu J., Tang Z., Yang G. (2025). From challenges and pitfalls to recommendations and opportunities: Implementing federated learning in healthcare. Med. Image Anal..

[208] Excedr Mass Spectrometer Costs: How Much Should You Budget?. 2025..

[209] National Academies of Sciences, Engineering, and Medicine, Health and Medicine Division, Board on Population Health and Public Health Practice, Committee on the Long-Term Health and Economic Effects of Antimicrobial Resistance in the United States (2022). Combating Antimicrobial Resistance and Protecting the Miracle of Modern Medicine.

[210] Tsitou V.-M., Rallis D., Tsekova M., Yanev N. (2024). Microbiology in the era of artificial intelligence: Transforming medical and pharmaceutical microbiology. Biotechnol. Biotechnol. Equip..

[211] Paul S., Singh S., Chakrabarti A., Rudramurthy S.M., Ghosh A.K. (2019). Stable isotope labelling: An approach for MALDI–TOF MS-based rapid detection of fluconazole resistance in Candida tropicalis. J. Antimicrob. Chemother..

[212] Weis C.V., Jutzeler C.R., Borgwardt K. (2020). Machine learning for microbial identification and antimicrobial susceptibility testing on MALDI–TOF mass spectra: A systematic review. Clin. Microbiol. Infect..

[213] Wang J., Wang H., Cai K., Yu P., Liu Y., Zhao G., Chen R., Xu R., Yu M. (2021). Evaluation of three sample preparation methods for the identification of clinical strains by using two MALDI-TOF MS systems. J. Mass. Spectrom..

[214] Sandrin T.R., Goldstein J.E., Schumaker S. (2013). MALDI TOF MS profiling of bacteria at the strain level: A review. Mass. Spectrom. Rev..

[215] Topić Popović N., Kazazić S.P., Bojanić K., Strunjak-Perović I., Čož-Rakovac R. (2023). Sample preparation and culture condition effects on MALDI-TOF MS identification of bacteria: A review. Mass. Spectrom. Rev..

